# Evaluation of tractography parameters for dentato-rubro-thalamic tract reconstruction during pediatric posterior fossa tumor surgery

**DOI:** 10.1007/s10334-025-01297-5

**Published:** 2025-09-30

**Authors:** Pien E. J. Jellema, Karina J. Kersbergen, Eelco W. Hoving, Maarten H. Lequin, Kirsten M. van Baarsen, Thomas Lindner, Wouter P. Nieuwenhuis, Alberto De Luca, Jannie P. Wijnen

**Affiliations:** 1 Department of Pediatric Neuro-Oncology, Princess Máxima Center for Pediatric Oncology, Utrecht, The Netherlands; 2 Centre for Image Sciences, University Medical Center Utrecht, Utrecht, The Netherlands; 3 Edward B. Singleton Department of Radiology, Texas Children’s Hospital, Austin, TX USA; 4 Department of Diagnostic and Interventional Neuroradiology, University Hospital Hamburg-Eppendorf, Hamburg, Germany; 5 Department of Radiology, University Medical Center Utrecht, Utrecht, The Netherlands; 6 Department of Neurology, University Medical Center Utrecht, Utrecht, The Netherlands

**Keywords:** Diffusion tractography, Cerebellar white matter, Pediatrics, Posterior fossa tumor, Neurosurgery

## Abstract

**Objective:**

Intraoperative fiber tractography can help neurosurgeons in localizing eloquent tracts potentially displaced by tumors. In pediatric posterior fossa tumor (pPFT) patients, disturbances to the eloquent dentato-rubro-thalamic tract (DRTT) might contribute to cerebellar mutism syndrome. This study investigates the effect of fiber tractography parameters on DRTT reconstruction in pre- and intraoperative settings.

**Methods:**

T1-weighted and diffusion MRI data were acquired from ten pPFT patients and two healthy volunteers. The patients were scanned pre- and intraoperatively. The DRTT was reconstructed using multiple fiber orientation distribution (FOD) and angle thresholds. An expert panel evaluated tract reconstructions to identify optimal parameters in our dataset. The corticospinal tract (CST) served as a control. Relative tract volumes of the DRTT and the CST were calculated.

**Results:**

Diffusion MRI data were sufficient for reliable DRTT reconstruction in healthy volunteers. In most pPFT patients, an FOD of 0.01 and a 60° angle threshold were evaluated as optimal for DRTT reconstruction in our dataset. Preoperative DRTT reconstructions showed more reconstructed streamlines and larger relative volumes, particularly in non-decussating tracts. CST reconstructions remained consistent across both timepoints.

**Discussion:**

DRTT reconstruction is feasible in pPFT patients before and during surgery. However, inter-subject variability suggests that some patients may require adjusted thresholds for optimal results.

**Supplementary Information:**

The online version contains supplementary material available at 10.1007/s10334-025-01297-5.

## Introduction

**Fig. 1:**
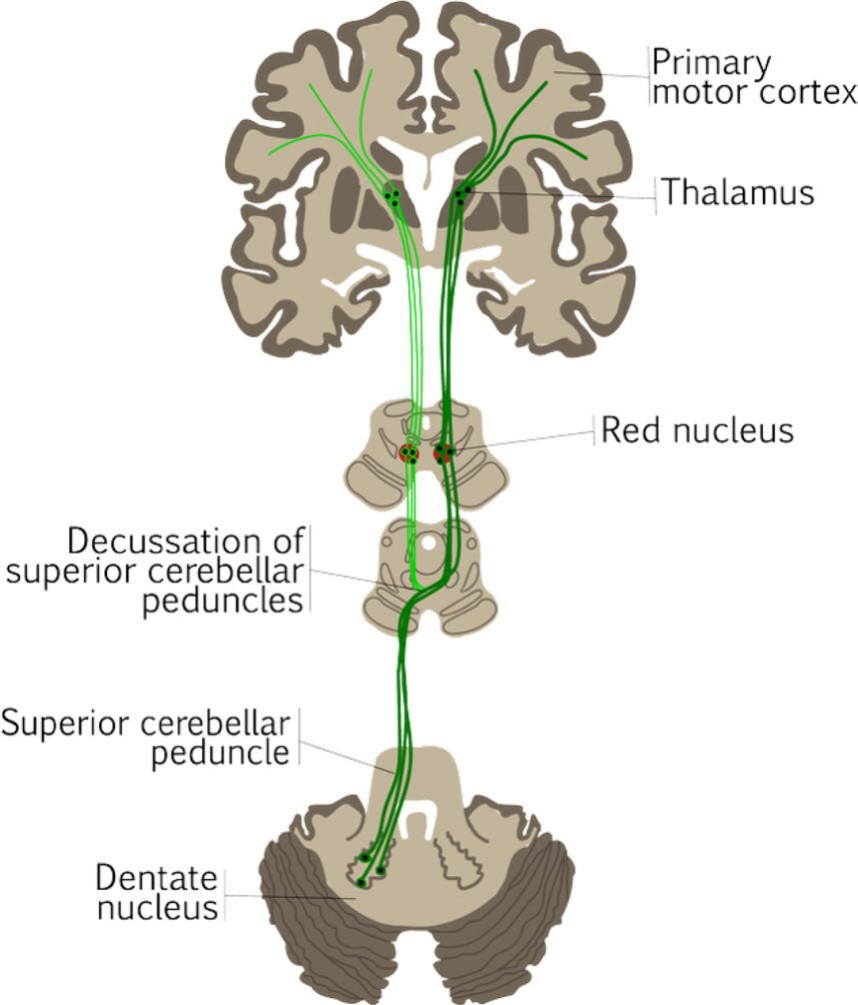
Anatomical illustration of the dentato-rubro-thalamic tract (DRTT). The dark green tract represents the decussating DRTT, the classic component that decussates from the dentate nucleus to the thalamus and primary motor cortex in the contralateral hemisphere. The light green tract illustrates the non-decussating DRTT, a component that remains in the same hemisphere

Fiber tractography, based on diffusion MRI (dMRI) data, can help neurosurgeons identify eloquent white matter tracts during preoperative planning. During neurosurgical resection of brain tumors, a combination of loss of cerebrospinal fluid and dissection of brain tissue will create brain shift [1]. This can affect the accuracy of using preoperative fiber reconstructions during surgery. This inaccuracy may further be increased by mass reduction of the tumor. Therefore, an intraoperative update of the fiber tractography could potentially restore the accuracy of the eloquent tract reconstructions [2]. In the case of pediatric posterior fossa tumor (pPFT) patients, cerebellar mutism syndrome (CMS) can occur after surgical resection. The exact pathogenesis of CMS has not been elucidated yet, but it is thought to be related to a disturbance of the dentato-rubro-thalamic tract (DRTT) [3]. The decussating component of the DRTT (d-DRTT) connects the cerebellum to the cerebral cortex, originating in the dentate nucleus. It travels through the superior cerebellar peduncle and decussation in the brainstem to the contralateral red nucleus, proceeds to the thalamus, and finally projects on the primary motor cortex (Fig. 1) [4]. In addition, a non-decussating (nd-DRTT) component connects the dentate nucleus to the red nucleus and thalamus within the same hemisphere [5, 6]. The DRTT is responsible for coordinating complex movements and cognitive functions like verbal communication, planning, and working memory [7, 8]. Damage to the DRTT during surgery can lead to deficits in these brain functions, manifesting as CMS symptoms [9]. Intraoperative reconstruction of the DRTT could provide actual information about the integrity of the DRTT potentially affected by tumor resection or re-expansion of the surrounding structure like the cerebellar peduncles. Evaluation of intraoperative integrity changes to the DRTT might contribute to a better understanding of the pathogenesis of CMS [10].

Reconstructing the DRTT from intraoperative data in pPFT patients is challenging due to several factors. First, the acquired dMRI data are of limited quality, characterized by lower signal-to-noise ratio (SNR) and more imaging artifacts due to the surgical cavity. These technical limitations can be attributed to time constraints and fewer RF receive coils. Second, strong brain deformations caused by brain shift, displacement by tumor tissue, and additionally the presence of hydrocephalus may affect or complicate the DRTT reconstruction. Next to technical challenges, it has been shown that the DRTT has a high inter-subject anatomical variability and follows a complex trajectory with high curvatures and crossings (as in the decussation) [4]. This makes it more challenging to reconstruct the DRTT compared to other eloquent tracts such as the corticospinal tract which is more consistent in shape across subjects. Previous research has attempted to clarify the neuroanatomy of the DRTT in adults using various methods, such as creating a dMRI based atlas [11], post-mortem white matter dissection [12], or comparing post-mortem fiber tractography with a 3D histological reconstruction [13]. Another approach involved deterministic fiber tractography applied to a large dataset of healthy adults [5], which is a method that reconstructs a single fiber trajectory (streamline) from a seed point based on the principal diffusion direction [14, 15]. Lipp et al. (2022) [16] also used deterministic fiber tractography to study the DRTT anatomy. They focused on younger Parkinson’s patients (average age 25) due to the clinical correlation of DRTT impairment and tremor symptoms [16, 17]. Despite enough evidence on DRTT anatomy in various adult populations, a fiber tractography protocol is missing for the pediatric brain tumor population. In particular, a protocol optimized for both pre- and intraoperative use in this population remains unavailable.

In this study, the goal is to determine the best combination of parameters for deterministic fiber tractography in our dataset, including angle and fiber orientation distribution thresholds, that accurately represent the DRTTs neuroanatomy in pPFT patients before and during posterior fossa tumor surgery.

## Methods

Given the absence of a gold standard for the pPFT population, we validated our tract reconstructions through a qualitative assessment by a panel of neuroanatomical experts. As a quantitative measure, we calculated the relative tract volume to whole brain white matter. The dMRI data used for the fiber tractography were obtained from our institution’s protocol optimized for intraoperative MRI (ioMRI) acquisition [18]. Data from healthy volunteers were included to assess whether our intraoperative dMRI protocol is of sufficient quality to reconstruct tracts in a healthy brain. We also examined whether a control tract outside the resection area maintained consistent reconstructions between the pre- and intraoperative acquisitions.

### Study population

Ten pPFT patients (aged 3–17 years; four females) and two healthy volunteers (aged 25–27 years; one female) were included in this study (Table 1). The local ethics committee approved this study. All subjects and/or caregivers provided written informed consent.

**Table 1:** Overview of demographic details of healthy volunteers and patients included in our study

	Sex assigned at birth	Age at date of MRI	Tumor location	Tumor type	Prior resection (Y/N)	Extent of resection
Healthy volunteers						
1	M	25	–	–	–	–
2	F	27	–	–	–	–
Patients						
1	M	3	Right peduncle	Pilocytic astrocytoma	No	Partial
2	F	14	4th ventricle	Medulloblastoma	No	Complete
3	F	12	4th ventricle	Pilocytic astrocytoma	No	Partial
4	F	3	Midline tumor/Left mesencephalon	Pilocytic astrocytoma	No	Partial
5	M	11	4th ventricle right	Pilocytic astrocytoma	Yes	Complete
6	M	9	Right cerebellopontine angle	Pilocytic astrocytoma	Yes	Complete
7	M	6	4th ventricle	Ependymoma	No	Complete
8	M	17	4th ventricle	Medulloblastoma	No	Complete
9	F	6	Cranial in the vermis	BCOR Sarcoma	Yes	Partial
10	M	11	4th ventricle	Medulloblastoma	No	Complete

*BCOR* BCL-6 transcriptional corepressor

### Experimental setup

**Fig. 2:**
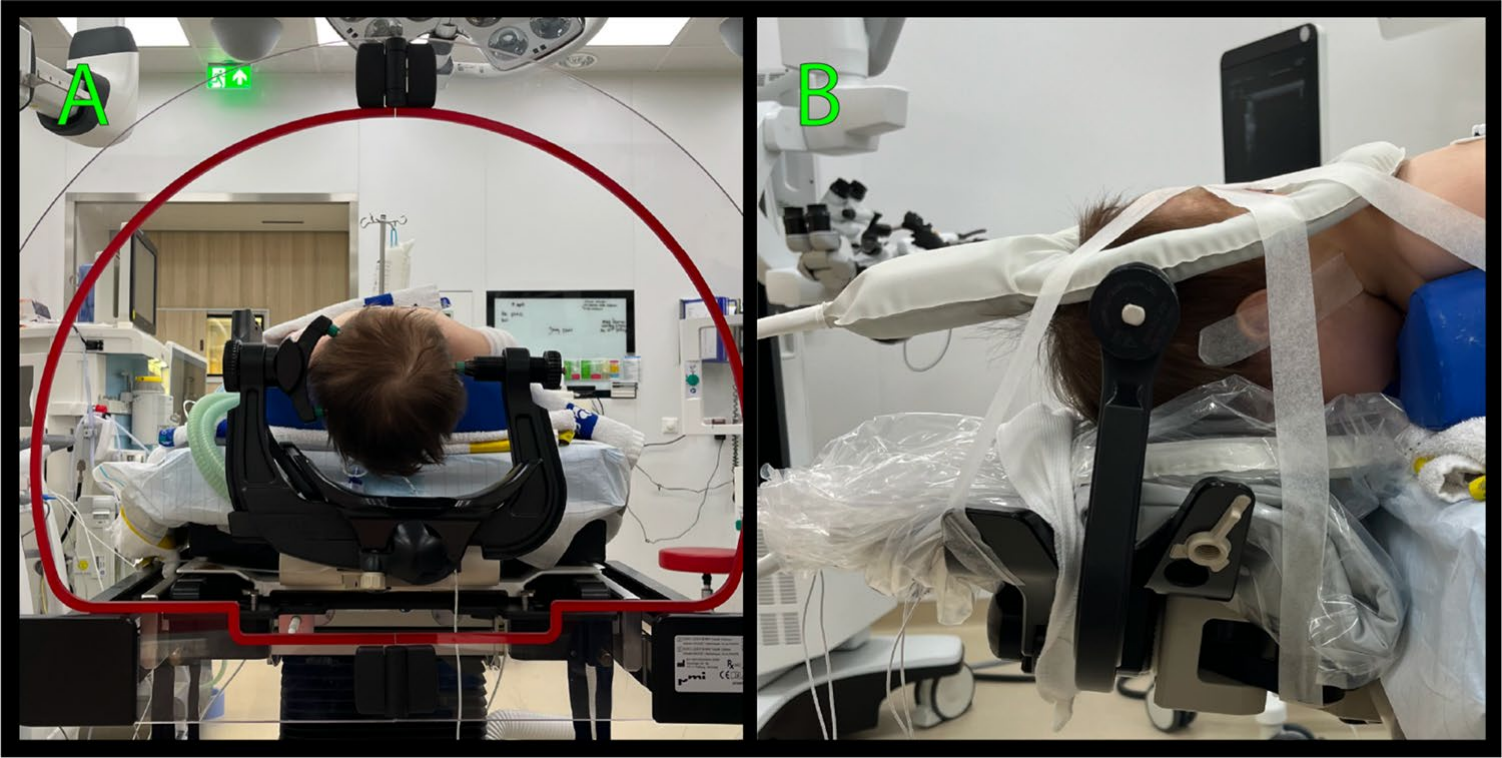
Example of patient preparation for MR acquisition before surgery. **A** After positioning the patient in the surgical prone position (i.e., laying on their stomach with their chest lifted and chin-down), the head is secured in the DORO surgical head-frame. **B** The two RF coils are then placed anterior and posterior of the patients’ head and fixated with tape before sliding the patient into the intraoperative MRI scanner that is in a room adjacent to the operation theater

**Fig. 3:**
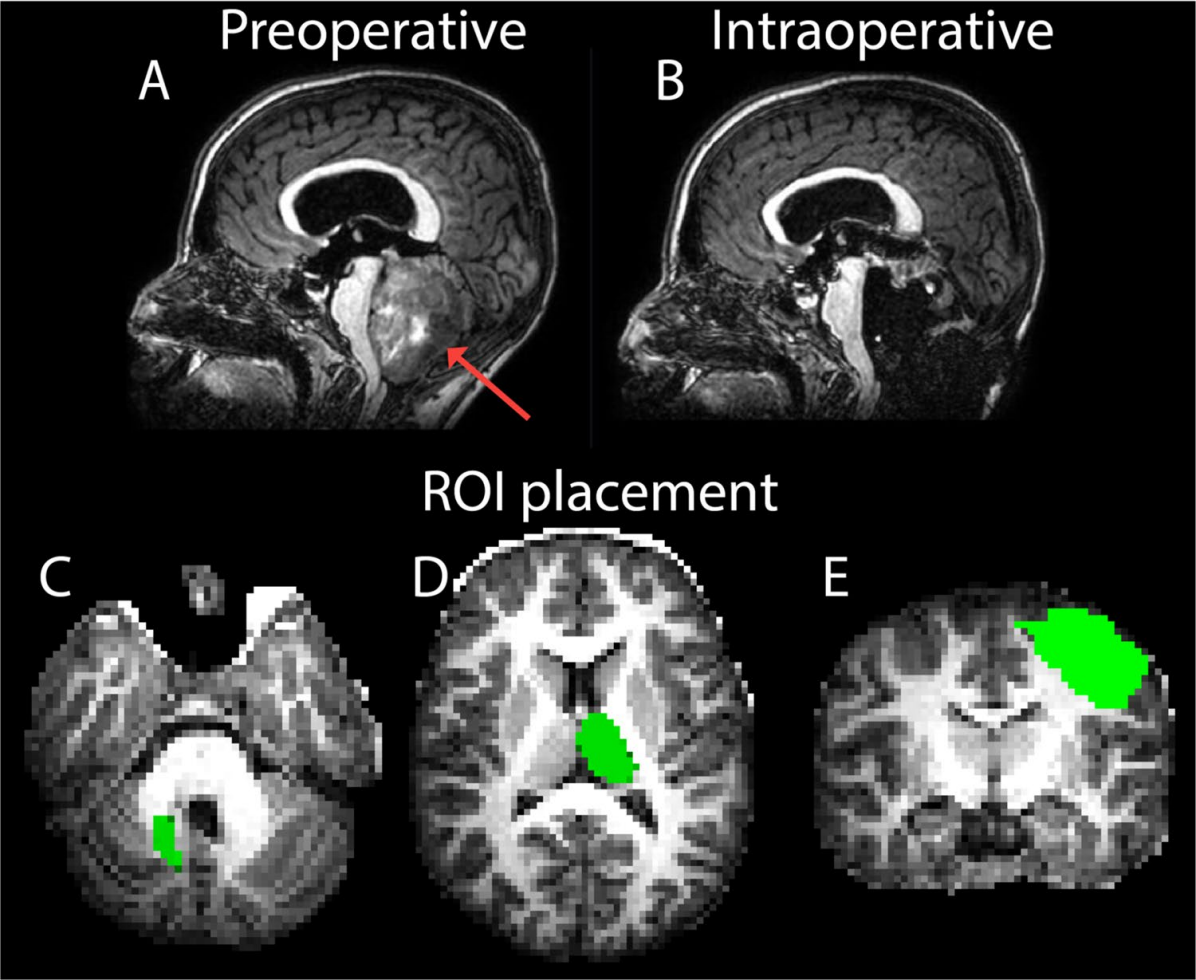
Example of T1-weighted images before and during surgery and placement of the regions of interest used for fiber tractography. T1-weighted (T1w) images of a 14-year-old girl with a 4th ventricle tumor before resection (**A**, preoperative) and after surgical resection but before closure of the skull (**B**, intraoperative). The red arrow in A indicates the tumor in situ. To reconstruct the dentato-rubro-thalamic tract, the dentate nucleus (**C**), thalamus (**D**), and primary motor cortex (**E**) were included as regions of interest (ROIs), indicated in green. For detailed segmentation results see appendix 3, Fig. 1 (Supplementary material)

We obtained all MR images of the pPFT patients with a 3-Tesla intraoperative MRI scanner (Philips Ingenia ElitionX MR-OR system, 70 cm bore, Philips Healthcare, Best, The Netherlands) with two RF coils (Fig. 2b). Both pre- and intraoperative images (Fig. 3a, b) were acquired on the day of surgery under the same anesthesia with the patient in a prone position (i.e., lying on their stomach with their chest lifted and chin-down), and the head secured in the DORO head clamp (Black Forest Medical Group, Fig. 2a). The preoperative scan is acquired before craniotomy. The intraoperative scan is acquired with an open skull at the time when most of the tumor is resected and the neurosurgeon decides to check for residual tumor tissue. If there is any remnant tumor visible on the intraoperative scan, the neurosurgeon has the option to continue to resect additional tumor tissue. MR images of the healthy volunteers were obtained with another 3-Tesla MRI scanner (Ingenia, 70 cm bore, Philips Healthcare, Best, The Netherlands) using the same two RF coils. Healthy volunteers were scanned once in the supine position (i.e., lying on their back with their head in a neutral position) without anesthetics or the head clamp.

### Imaging protocol

The scan protocol consisted of a T1-weighted (T1w) and three dMRI acquisitions (Table 2). The T1w images were used for registration and region of interest (ROI) segmentation purposes. The dMRI acquisition was a spin-echo EPI using Stejskal-Tanner gradients. The first dMRI acquisition contains one non-weighted diffusion image (b = 0 s/mm^2^, b0 image) and twenty images with different diffusion directions at a b-value of 1000 s/mm^2^ (b1000). This acquisition is sent to the clinical PACS system as a standard diffusion tensor image (DTI) series. The second acquisition completes the multi-shell dMRI dataset and contains five b0 and 32 images with different diffusion directions at a b-value of 2000s/mm^2^ (b2000). The gradient directions were optimized for the second shell on a half sphere using QMRITools [19]. For the first shell, we had to rely on the predefined gradient table from Philips to ensure compatibility with the neuronavigation system (Brainlab, Munich, Germany). The optimization of this gradient table was done by electrostatic repulsion on half a sphere with antiparallel gradients. In healthy volunteers, susceptibility distortions were partly corrected by affine registration to the T1w image [20]. In patients, we added a third acquisition with reversed phase encoding direction settings to correct for the susceptibility distortions that are expected during the ioMRI acquisition using FSL topup [20]. The time of the three dMRI acquisitions was ~ 9 min and has been optimized for the surgical setting [18]. The repetition time (TR) was set on a range from 7000 to 8200 ms for all dMRI acquisitions to allow for variability in head orientation and to ensure thermal safety. Certain head angulations can lead to increased gradient load, and the system automatically adjusts the TR within the specified range to prevent overheating. However, as can be seen in Appendix 7 (Supplementary material), the TR values for all patients and measurements in the study varied very little; all TR values were between 7720 and 7734 ms.

**Table 2:** Imaging parameters

Sequence	T1wHealthy volunteer	Patient	dMRI		
			1) B1000	2) B2000	3) Rev. enc
Field of view (mm)	351 × 251x160	230 × 185x160	240 × 240x150	240 × 240x150	240 × 240x150
Acquired voxel size (mm)	1.2 × 1.2x1.2	1.25 × 1.25x1.25	2.5 × 2.5x2.5	2.5 × 2.5x2.5	2.5 × 2.5x2.5
Echo time (ms)	2.1	3.0	99	99	99
Repetition time (ms)	4.7	6.5	7000—8200	7000—8200	7000—8200
[G, δ, Δ] [(mT/m), (ms), (ms)]	–	–	[15.9,38.9, 49.5]	[22.5, 38.9, 49.5]	[15.9, 38.9, 49.5]
Acceleration	CS 1.8	CS 1.2	S 1.8	S 1.8	S 1.8
Acquisition time	3 min. 52		3 min	5 min	1 min
Diffusion encoding (s/mm^2^)	NA	NA	1 × b = 0, 20 × b = 1000	5 × b = 0, 32 × b = 2000	1 × b = 0, 6 × b = 1000
Phase encoding direction	RL	RL	AP	AP	PA
Imaging mode	3D multishot TFE	MPRAGE	2D Single shot EPI	2D Single shot EPI EPI	2D Single shot EPI

The T1-weighted (T1w) acquisition of the healthy volunteers was based on the original clinical protocol. For the intraoperative acquisition of the patients, we aimed to increase the gray-white matter contrast of the T1w image and therefore used an MPRAGE acquisition. The first diffusion MRI (dMRI) acquisition is separated to send through to the clinical PACS system. The second acquisition completes the multi-shell dMRI dataset. The third acquisition is acquired only in patients, with reverse phase encoding (rev. enc.) direction settings to correct for the echo planar imaging (EPI) distortions. G = gradient amplitude, δ = gradient duration,Δ = temporal separation, S = SENSE, CS = compressed SENSE, AP = anterior–posterior, PA = posterior-anterior. TFE = turbo field echo, MPRAGE = magnetization prepared rapid gradient echoImaging

### Region of interest segmentation

We segmented brain regions that are used as either inclusion regions, through which a streamline must pass (AND), and exclusion regions through which streamlines cannot pass (NOT). To reconstruct the DRTT, we used the dentate nucleus, thalamus, and primary motor cortex as inclusion regions and the corpus callosum and preoperative tumor volume as exclusion regions [4]. All regions were segmented based on the T1w images. Automatic segmentation of these regions, especially the dentate nucleus, was challenging due to the strong brain deformations that result from the preoperative tumor or intraoperative resection cavity. To address this, we performed separate segmentations on the pre- and intraoperative datasets, avoiding misregistration across sessions. Whole-brain gray and white matter masks were generated using the online brain segmentation pipeline of volBrain [21] combined with the CERES pipeline for improved detail of the cerebellum [22]. Thalamus segmentations were retrieved from the volBrain pipeline. We then registered the whole brain gray and white matter masks to the SUIT template [23]. The resulting registration matrix was then used to register the SUIT cerebellar atlas, which included the left and right dentate nuclei [24], back to the native gray and white matter masks (see appendix 3 in Supplementary material for detailed segmentation results). Tumor tissue of the pPFT patients was semi-manually segmented using the Smart Brush tool in the Brainlab neuronavigation system (Smart Brush, Brainlab, Munich, Germany). The anatomical accuracy of the dentate nucleus and tumor volume were verified and adjusted by a pediatric neuroradiologist (W.N., 5 years of experience), using both T1w and T2-weighted (T2w) scans available in the patient archiving system. The T2w images offer improved visibility of the dentate nucleus [25]. The T1w image was linearly registered to the dMRI data using an affine transformation and was then used for the co-registration of the ROIs to subsequently use them in the fiber tractography pipeline (Fig. 3c, d). The primary motor cortex was directly registered from the Desikan atlas [26] to the dMRI data, and the corpus callosum from the Destrieux atlas [27] in a similar way.

To reconstruct the corticospinal tract (CST) as a control tract outside the resection area, we included the primary motor cortex and brainstem regions. Exclusion regions for the CST were the corpus callosum, thalamus, cerebellum, contralateral hemisphere white matter, superior frontal gyrus, and occipital and temporal lobes (all regions derived from the Desikan atlas [4, 26]. For pPFT patients, preoperative tumor tissue was additionally used as an exclusion region for preoperative CST reconstruction.

### Diffusion MRI preprocessing and fiber tractography

Data of the dMRI acquisitions were used to reconstruct the DRTT and CST. Preprocessing was done with the fully automated MRIToolkit pipeline (https://github.com/delucaal/MRIToolkit.git) and ExploreDTI [28, 29]. Data preprocessing involved correcting for signal drift [30], denoising with the Marchenko-Pastur Principal Component Analysis (MPPCA) [31], correction for eddy currents and subject motion utilizing affine registration, and b-matrix correction [29]. Brain masking was done with the Brain Extraction (BET) FSL toolbox [32]. We corrected susceptibility distortions by means of the reversed phase encoding direction data using FSL topup [20]. After preprocessing, the DKI model was fitted [33, 34], as in our previous work [35]. Given that DKI is prone to spurious mean kurtosis (MK) values, we employed the MK-curve correction method [33, 34]. The Generalized Richardson Lucy spherical deconvolution method [28] was applied to reconstruct fiber orientation distributions in white matter while accounting for partial volume with gray matter and cerebrospinal fluid.

The tractography analysis was performed in two steps. In the first step, we aimed to optimize the tracking parameters specifically for reconstructing the DRTT in our dataset. To this end, we seeded from the dentate nucleus and the thalamus. In the second step, we applied the optimized parameters identified in step one to perform whole-brain seeding. This allowed us to reconstruct control tracts located outside the DRTT region.

To identify the best deterministic fiber tractography parameters in our dataset, we created eight different combinations of parameter combinations varying the angle threshold (45° or 60°) and the fiber orientation distribution (FOD) amplitude threshold (0.1, 0.05, 0.01, or 0.005). Streamlines were terminated if they changed direction by an angle exceeding the angle threshold or if the local FOD amplitude was below the FOD threshold. Tractography was performed using deterministic FACT propagation (ExploreDTI, [29] with seed points placed every 0.5 mm isotropically. The step size was fixed at 1 mm for all experiments, given its intrinsic relation with the angle threshold. For parameter optimization, streamlines were seeded from the dentate nucleus and contralateral thalamus (d-DRTT), or ipsilateral thalamus (nd-DRTT). We then gated the tracts through the inclusion and exclusion regions defined in section “Region of interest segmentation”.

Given the lack of a gold standard, such as postmortem tractography or intraoperative electrical stimulation, we validated the tracts by consulting an anatomical expert panel to identify the parameter combination that aligns best with the neuroanatomy of the DRTT. This qualitative assessment has been detailed in paragraph 2.6.

After determining the best deterministic fiber tractography parameters for DRTT reconstruction in our dataset, we repeated the tractography using whole-brain seeding to enable reconstruction of control tracts beyond the DRTT region. To contain data size, the spacing between seed points was set to 1 mm isotropic. Streamlines were terminated at the gray-white matter interface. For the final DRTT and CST reconstructions, we gated through the above-mentioned (paragraph 2.4) inclusion and exclusion regions.

For quantitative analysis of the tracts, we computed the volume of the pre- and intraoperative DRTT and CST by counting the voxels that contain at least one streamline normalized to the whole brain white matter volume. The tract volumes of the pre- and intraoperative data are summarized in a violin/boxplot format, generated using the default settings in R’s ggplot2 [36]. The violin plots show a smoothed estimate of the data distribution, derived with a Gaussian kernel density estimator (frequency histogram) [37]. Differences in relative tract volumes of patients were analyzed by means of a paired Wilcoxon signed-rank test (significance level *p* < 0.05). To generate an SNR map, we divided the mean of the signal intensity for each voxel of all b0 images by their standard deviation. We then computed the average SNR across all voxels within the white matter mask.

## Qualitative analysis

**Fig. 4:**
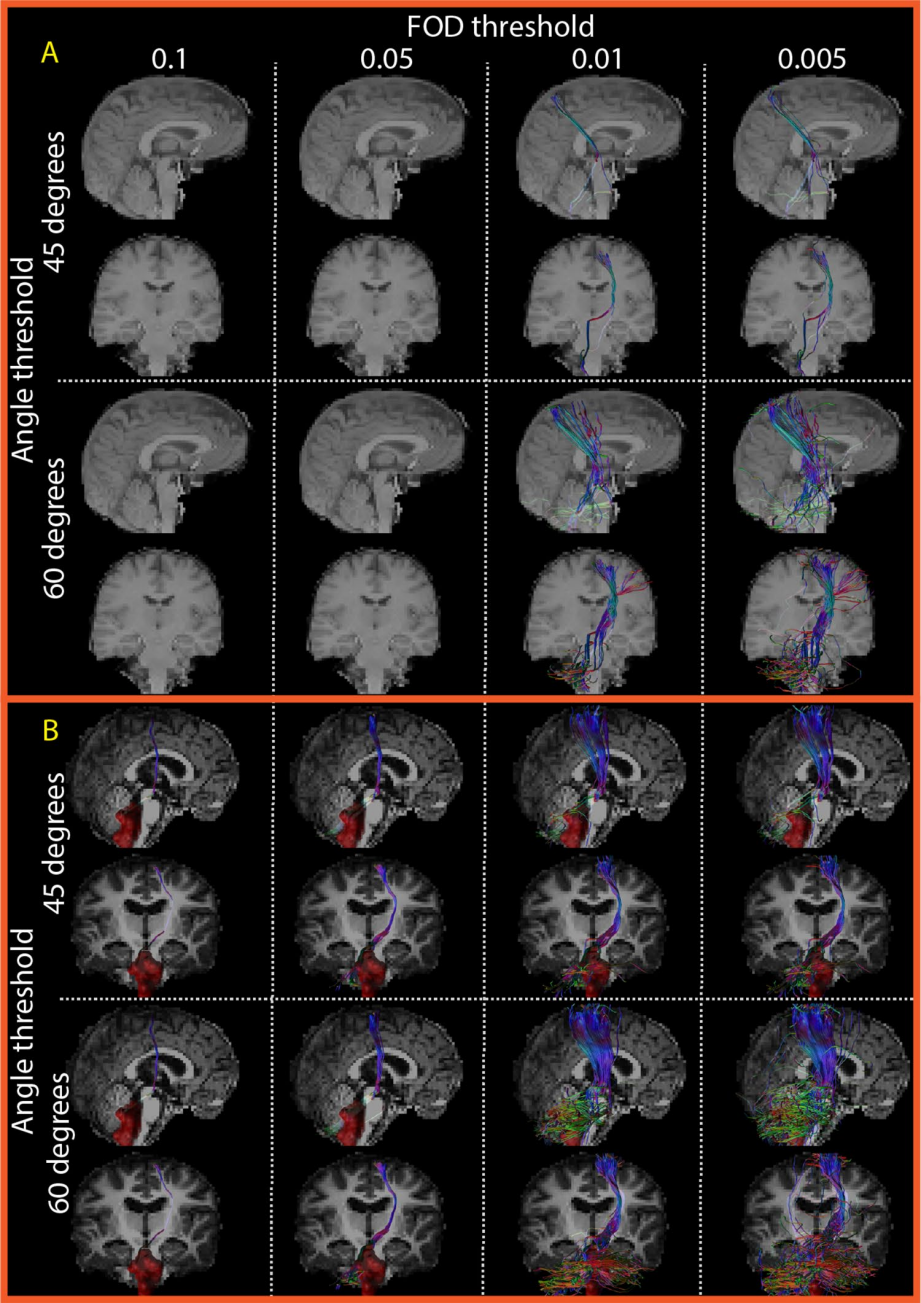
Example of eight fiber tractography parameter combinations for one side dentato-rubro-thalamic tract. All panels show the dentato-rubro-thalamic tract (DRTT) crossing from the dentate nucleus to the contralateral hemisphere. The upper panel displays data from a healthy volunteer, and the lower panel shows preoperative results for an example patient. More liberal settings increased visual coherence of streamlines but introduced false positives, as seen with an FOD threshold of 0.005 and an angle threshold of 60°. Parameter combinations were presented in a randomized order and blinded for the panel. A different T1-weighted contrast was used for healthy volunteers and patients

The anatomical expert panel (pediatric neurologist K.J.K. with 4 years of experience and pediatric neurosurgeon E.W.H. with 25 years of experience) identified the parameter combinations that aligned best with the neuroanatomy of the DRTT. This was done by rating the results of eight sets of parameter combinations for both the left and right d-DRTTs and nd-DRTTs. Examples of these eight sets are shown in Fig. 4. The results for each set of parameter combinations were displayed in a randomized order, with the reconstruction parameters undisclosed to the panel. The rating was first done on data from healthy volunteers to control whether our limited data, feasible to acquire during surgery, would yield any tracts at all. We then repeated this process for the data of the pPFT patients. Assessment of the best result for a set of parameter combinations in our dataset was based on concordance with neuroanatomy (i.e., best correspondence to known anatomical landmarks or structures) [4, 11–13] and trustworthiness (i.e., the optimal balance between false positive and negative reconstructed streamlines). Scoring criteria of the qualitative analysis are shown in appendix 4 (Supplementary material). The overall most trustworthy DRTT reconstruction, for each subject, was also rated for overall morphological quality in the context of its relevance to neurosurgical planning, using a 10-point scale from 1 (very poor) to 10 (excellent). Finally, the frequency of parameter combinations producing the best results was calculated across the entire study population to identify the best settings.

## Results

### SNR

The mean SNR in the white matter of the healthy volunteers was 20 ± 2.3. The mean SNR of the preoperative data of the pPFT patients was 22 ± 5.6, and 22 ± 4.2 for the intraoperative data.

### Qualitative analysis

**Fig. 5:**
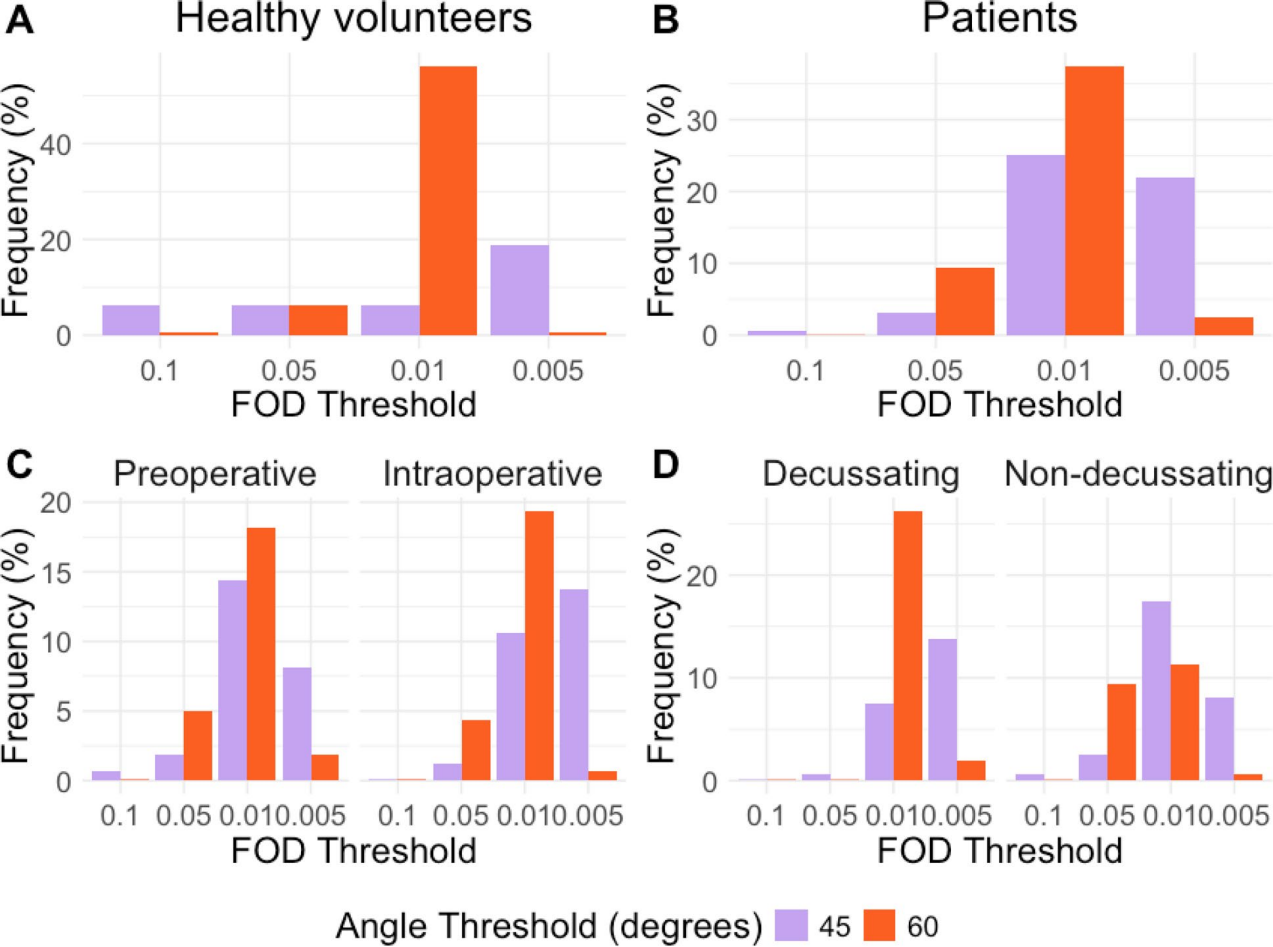
Qualitative results fiber tractography parameter combinations. In healthy volunteers (**A**) and pediatric posterior fossa tumor patients (**B**), an FOD threshold of 0.01 and an angle threshold of 60° were most frequently selected. This is still evident in the pre- and intraoperative results of the tractography (**C**). However, for the non-decussating data (**D**), an FOD of 0.01 and a narrower angle threshold of 45° were preferred. Every reconstructed tract was evaluated independently by each member of the neuroanatomical expert panel

The results for a parameter combination of an FOD threshold of 0.01 and an angle threshold of 60° were preferred most frequently by the anatomical expert panel for healthy volunteers and most pPFT patients (Fig. 5a, b). This was similar for the pre- and intraoperative datasets of the pPFT patients (Fig. 5c). When focusing on the d-DRTT reconstructions solely, again an FOD threshold of 0.01 and an angle threshold of 60° were most frequently chosen (Fig. 5d). However, for the nd-DRTT reconstructions, an angle threshold of 45 was favored by the neuroanatomical expert panel (Fig. 5d). The quality of the DRTT reconstructions with the best parameter set (FOD threshold = 0.01, angle threshold = 60°) was rated as 6.8 ± 1.2 in the context of its relevance to neurosurgical planning. The panel noticed in some pPFT patients false positive streamlines that ran through the corpus callosum.

The anatomical experts agreed in 25% of the cases on their preferred parameter combination. In 11% of the cases, they preferred the same angle threshold (45°) but different FOD thresholds, specifically 0.01 or 0.005. Vice versa, in 16% of the cases, both raters agreed on the same FOD threshold (0.01) but preferred a different angle threshold (45° or 60°). In 17% of all cases, one rater preferred an angle threshold of 60° with an FOD threshold of 0.01, while the other preferred an angle threshold of 45° with an FOD threshold of 0.005. These cases occurred primarily in patient datasets, where both parameter combinations produced reconstructions of comparable quality (appendix 6 in Supplementary material).

### DRTT fiber tractography using best parameters

**Fig. 6:**
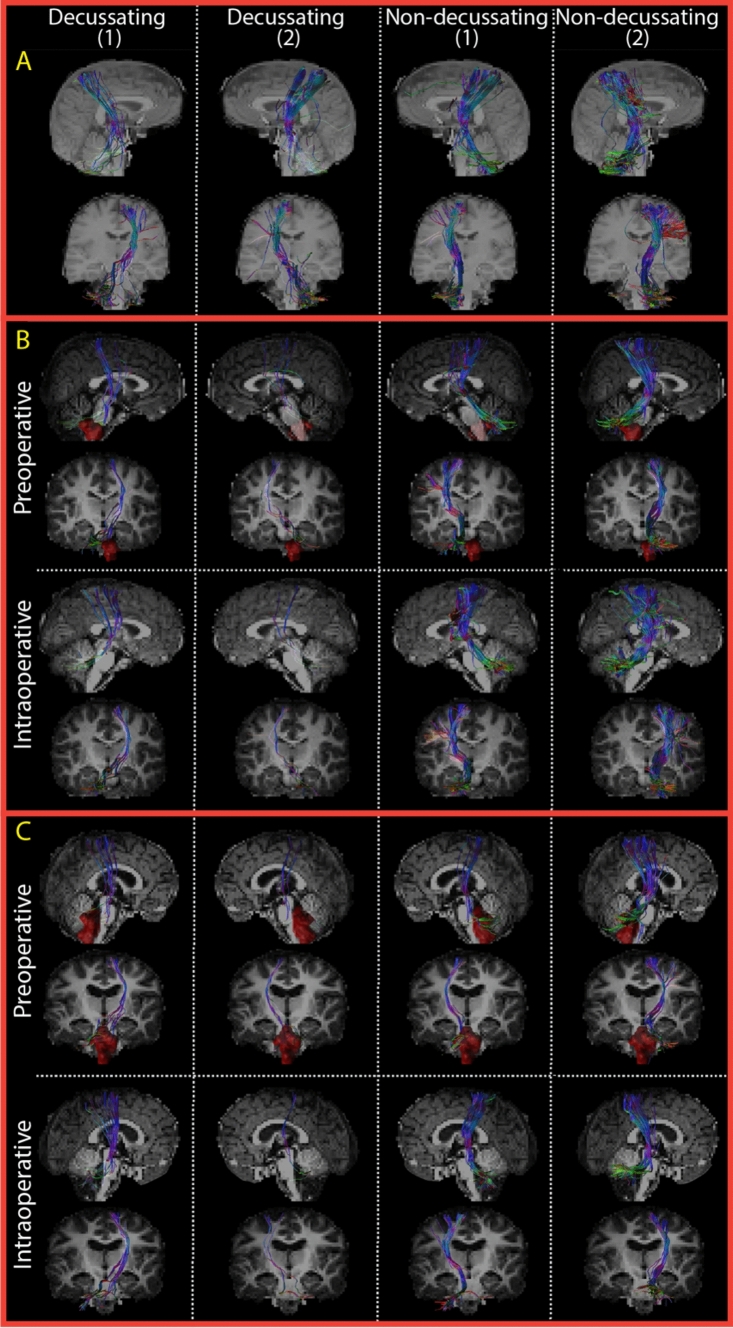
Dentato-rubro-thalamic tracts (DRTT) reconstructed with the best parameter combination of our dataset. The first two columns show both DRTTs (1 and 2) crossing from the dentate nucleus to the opposite hemisphere (decussating DRTT), while the last two columns show DRTTs staying on their original hemisphere (non-decussating DRTT). The example of a healthy volunteer (**A**) shows the DRTT with still some false positive streamlines present. In the examples of patients **B** and **C**, the decussating DRTTs show fewer streamlines and a less coherent bundle compared to their non-decussating ones. Within the patient examples, pre- and intraoperative reconstructions show similarly coherent bundles of streamlines. Preoperative tumors are indicated in red

The results of the repeated fiber tractography, performed after determining the best parameters in our dataset, are presented in Fig. 6. Although the number of streamlines has not been quantified, it can be visually observed that the pre- and intraoperative DRTT reconstructions show similarly coherent bundles of streamlines in some patients (Fig. 6). However, there appear to be more pPFT patients where the intraoperative DRTT is differently reconstructed with fewer streamlines and a less coherent bundle than the preoperative one. Furthermore, the nd-DRTT seems to be differently reconstructed with more coherent streamlines than the d-DRTT reconstruction in healthy volunteers and pPFT patients. In some cases, the chosen best settings for the population were too conservative to reconstruct any streamline of the DRTT. Specifically, reconstructions based on intraoperative data failed in 25% of the d-DRTT and 10% of the nd-DRTTs (data not shown). In the preoperative data, 5% of the d-DRTT reconstructions did not generate streamlines. These empty reconstructions were divided over four pPFT patients, including two who presented with hydrocephalus (pPFT pt 2 and 4, appendix 2 Figs. 3 and 4 in Supplementary material).

In a few pPFT cases, the quality of DRTT reconstruction improves following tumor resection (pPFT patients 8 and 9, appendix 2 Figs. 6 and 7 in Supplementary material). This improvement is particularly seen in patients with larger tumor volumes and is most pronounced on the side where the tumor caused more significant displacement.

### Control tracts

**Fig. 7:**
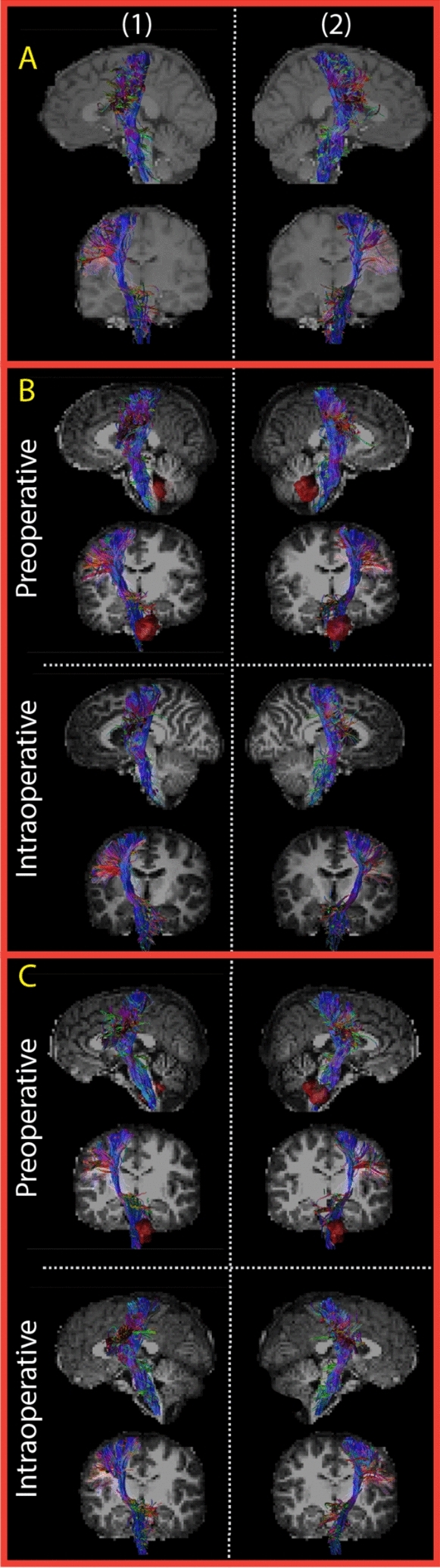
Corticospinal tracts (CST) reconstructed with the best parameter combination of our dataset. The example of a healthy volunteer (**A**) shows a sufficient visual coherence of streamlines on both sides of the CST (1 and 2). Pre- and intraoperative CST reconstructions are represented with similarly coherent bundle of streamlines in patients **B** and **C**. Preoperative tumors are indicated in red

Both sides of the CST were successfully reconstructed in all healthy volunteers and pPFT patients (Fig. 7). It can be visually observed that pre- and intraoperative CST reconstructions show similarly coherent bundles of streamlines in the pPFT population. In the same two pPFT patients presented with hydrocephalus (pPFT pt 2 and 4, appendix 2 Fig. 9 in Supplementary material,), the CST was reconstructed with fewer streamlines and a less coherent bundle.

### Relative tract volume

**Fig. 8:**
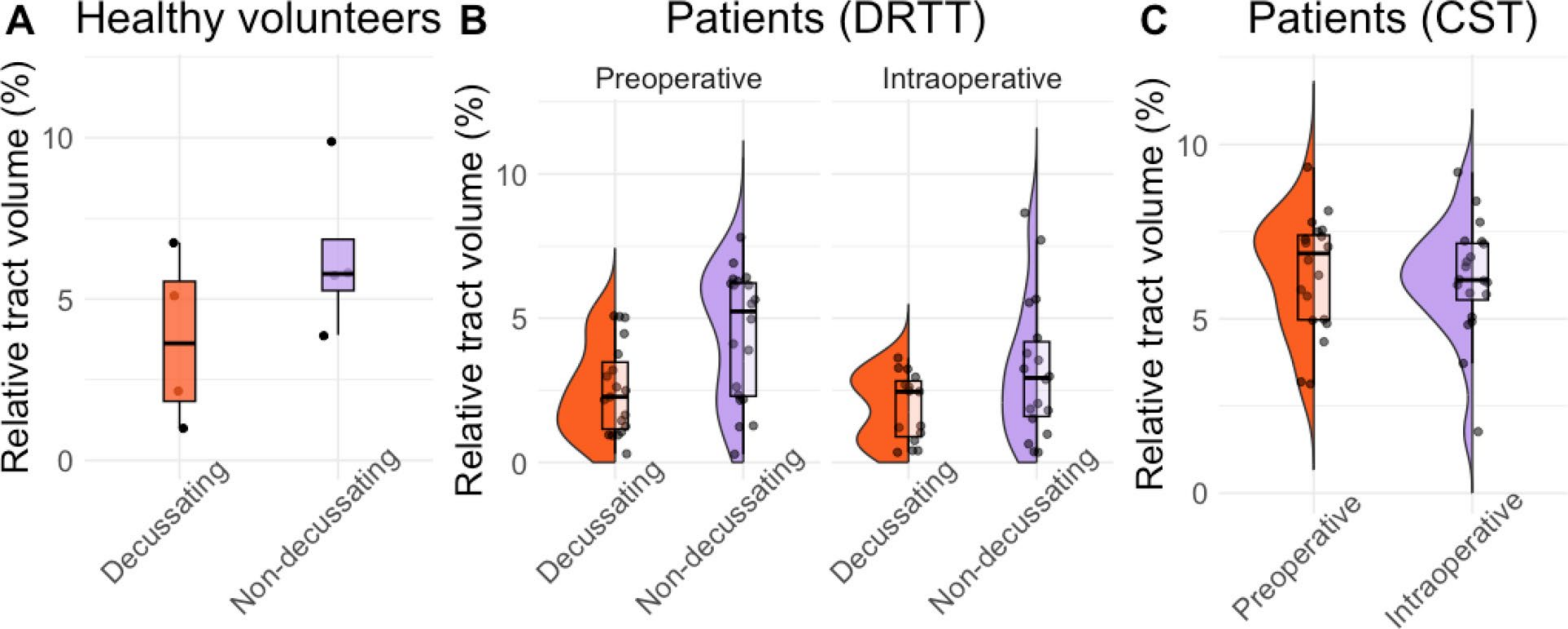
Tract volumes of the dentato-rubro-thalamic tracts (DRTT) and corticospinal tract (CST) reconstructed with the best parameter combination of our dataset. Tract volumes are expressed as percentages of the whole brain white matter volume. Each dot represents an individual subject measurement. The width of the violin at each y-value indicates the density of data points, with wider sections representing higher density. The bold horizontal line within the boxplot denotes the median, and the box itself represents the interquartile range (IQR), encompassing the 25th to 75th percentiles. In healthy volunteers (**A**) and preoperative patients (**B**), median tract volumes are lower for the decussating DRTT compared to non-decussating DRTT. Intraoperatively, DRTT components show similar volumes. Non-decussating tract volumes decrease from pre- to intraoperative sessions, while decussating volumes remain stable. CST volumes remain stable between pre- and intraoperative data (**C**)

The median relative volume was slightly higher in the nd-DRTT than in the d-DRTT reconstructions, in both healthy volunteer (Fig. 8a, nd-DRTT 5.8% [5.3–6.9], d-DRTT 3.6% [1.8–5.5]) and preoperative pPFT patients (Fig. 8b, nd-DRTT: 5.2% [2.3–6.2], d-DRTT 2.3% [1.2–3.5], *p* = 0.03). Intraoperatively, both DRTT components showed similar volumes (nd-DRTT 2.9% [1.6–4.2], d-DRTT 2.5% [0.9–2.8], *p* > 0.05). The relative volume of the nd-DRTT decreased significantly from the pre- to intraoperative session (2.3% difference, *p* = 0.01), while the d-DRTT volumes remained stable (*p* > 0.05). The violin plot of the preoperative nd-DRTT reconstructions showed two density peaks, one below and one above 5%. A post hoc analysis revealed that smaller preoperative nd-DRTT volumes were correlated with larger relative tumor volumes (*r* = − 0.61, *p* < 0.05, appendix 1 Fig. 3 in Supplementary material,).

As a control tract, the CST showed a median relative volume of 8.4% [7.8–9.1] in healthy volunteers. CST volumes were similar (*p* > 0.05) in the pre- (6.9% [5.0–7.4]) and intraoperative data (6.1% [5.6–7.2]) of pPFT patients.

## Discussion

We found that the dMRI data acquired with two RF coils during surgery was of sufficient quality for reliable DRTT reconstruction in healthy volunteers. When analyzing dMRI data from pPFT patients, in most cases the results for an FOD threshold of 0.01 and an angle threshold of 60° were rated as the best combination of parameters in our dataset to represent the DRTT’s neuroanatomy before and during surgery. The quality of DRTT reconstructions was generally higher in the preoperative data than in the intraoperative setting (with open skull). This was particularly evident for the nd-DRTT with a significant reduction in relative volume intraoperatively. Furthermore, the nd-DRTT was more robustly reconstructed than the d-DRTT, as reflected by a consistently higher relative volume and visual appearance of more coherent streamlines in preoperative pPFT patients. Pre- and intraoperative CST reconstructions showed similarly coherent bundle of streamlines and no significant relative volume differences.

Our finding that the acquired dMRI data were sufficient to reconstruct the DRTT in healthy volunteers aligns with our previous study [18], where we successfully reconstructed the CST and arcuate fasciculus using the same dMRI protocol. The best parameter combination identified for pPFT patients in this study is less conservative compared to studies conducted outside the surgical setting using head coils with more receive channels (e.g., with 32-channels). For example, Lipp et al. (2022) [16] used an FOD threshold of 0.05 and an angle threshold of 45° in younger Parkinson’s patients. However, their dMRI data acquisition lasted twice as long and used four times more RF channels, yielding a much higher SNR than is feasible intraoperatively. While other angle thresholds could have been tested, thresholds below 45° (e.g., 30°) likely increase false negatives due to the DRTT’s long, curved, narrow trajectory [4], while thresholds above 60° (e.g., 80°) may risk excessive false positives in our dataset [38, 39]. The overall trajectory and morphology of our reconstructed DRTT components are consistent with previous reports [5], particularly regarding the bifurcation into decussating and non-decussating pathways, as well as the decussation pattern in the midbrain. Intraoperative dMRI has inherently lower SNR than other research-oriented protocols. As such, our findings will likely not be optimal for higher SNR data. However, our findings can serve as a solid basis for future intraoperative studies.

The DRTT reconstructions appeared to have greater inter-subject variability than the CST [4]. Using the selected parameter combination, the visual coherence of streamlines in pPFT patients varied, with some reconstructions appearing sparse or even absent. Notably, reconstructions that were empty or had fewer streamlines and a less coherent bundle were observed in patients with hydrocephalus or brain shift (pPFT patients 2 and 4, appendix 2 Figs. 3 and 4 in Supplementary material,). These anatomical displacements likely affected the spatial alignment of inclusion and exclusion regions. Moreover, postoperative edema might partly include the trajectory of the DRTT. These two factors inherently lead to challenges for fiber tractography methods [40]. In such cases, more permissive thresholds may help preserve tracts that would otherwise not be reconstructed (appendix 5 in Supplementary material,). However, this would come at the price of many false positive streamlines that should be further removed. In contrast, stricter thresholds might improve anatomical specificity of the DRTT in patients with higher data quality and less anatomical deformations.

The Intraoperative DRTT showed fewer streamlines, a less coherent bundle, and a smaller volume than the preoperative ones. This reduction is likely due to intraoperative anatomical changes, particularly brain shift following tumor resection, which can alter the alignment between anatomical structures and tractography ROIs. These changes can result in fewer streamlines being reconstructed, even if the underlying tract remains intact. Differences in SNR did not account for the observed reduction in streamlines or the decreased coherence in bundles. Despite some challenges in aligning the RF coils intraoperatively on the sterile cloths that cover the resection cavity, comparable SNR was found in the pre- and intraoperative data. The CST, which is less susceptible to surgical manipulation and brain shift, maintained consistent tract volumes between both sessions.

We found a lower relative volume of the d-DRTT reconstructions compared to the nd-DRTT reconstruction. However, based on dissection evidence, the d-DRTT is expected to have a higher volume than the nd-DRTT [5]. Consistent with our fiber tractography results, Petersen et al. (2018) [6] also reported more nd-DRTT than d-DRTT streamlines that completed tracking. This discrepancy may be attributed to false-negative results of the tractography algorithm when reconstructing crossing fibers, particularly in regions where multiple fiber bundles intersect, such as in deep white matter [41]. To address this, we acquired multi-shell dMRI data, which is more suited for reconstruction of multiple fiber populations (e.g., crossing fibers) within a single voxel [42]. Still, false-negatives may persist due to limited spatial resolution (2.5 mm isotropic), which may not sufficiently differentiate the diffusion directions of distinct DRTT components within a single voxel. An underestimation of the d-DRTT volume could lead to inaccurate interpretations of the operative planning and evaluation. Moreover, given the volume difference between the d-DRTT and nd-DRTT, different tractography thresholds may be more optimal for each subtype. If the DRTT is the primary outcome of interest, higher spatial resolution should be considered to improve anatomical accuracy, though this would require longer acquisition times or improved coil hardware to maintain adequate SNR.

This study has some limitations. To begin with, we were unable to validate our results against a gold standard, such as a postmortem tractography or intraoperative electrical stimulation [13, 15]. Instead, anatomical accuracy was assessed by a panel of neuroanatomical experts using established histological, dissection, and tract atlas-based references [4, 11–13]. Consensus among the panel on the preferred parameter combination was limited, likely reflecting personal preference: one expert may have favored more streamlines and coherent bundles, tolerating more false positives, while the other may have preferred fewer streamlines with stricter adherence to neuroanatomical accuracy. Thus, depending on the clinical objective, either conservative or liberal thresholds could be appropriate. Another limitation is that we did not account for the effects of varying tumor types on the fiber tractography results. Larger tumor masses may cause greater fiber displacement, while tumors with surrounding edema or cysts could challenge the reliability of the tractography due to more isotropic diffusion [15]. Nonetheless, our objective was to define tractography parameters that are broadly applicable to the diverse range of tumors seen in pPFT patients. In addition, while some degree of misregistration between T1w and dMRI data is unavoidable, we used state-of-the-art susceptibility correction [20] and manually verified anatomical alignment for each pre- and intraoperative dataset. The use of multiple ROIs further increases the tractography robustness to local misalignments. Moreover, we only considered deterministic fiber tractography, which is known to be repeatable but also limited in mapping complex fiber geometries [43]. Nevertheless, the overall findings regarding parameter sensitivity, the trade-off between achieving more streamlines in a coherent bundle or prioritizing anatomical specificity, and the practical constraints of intraoperative acquisition are likely applicable to probabilistic approaches. The optimal parameters may be, however, different for these tractography approaches. We also note that only the primary motor cortex was considered as the thalamo-cortical endpoint of the DRTT [4, 8]. However, some studies indicate that the DRTT also projects to the premotor cortex and prefrontal cortex [44]. Including these additional regions in our tractography protocol could provide deeper insights into speech initiation and coordination distortions in children with CMS [45]. Nonetheless, in this exploratory study, we limited our focus to the primary motor cortex as the primary output region. Finally, the number of healthy volunteers was small, and their data were acquired under non-patient conditions. In practice, these datasets served only as a quality control of the evaluation pipeline, offering insight into tractography outcomes in brains unaffected by tumor mass or intraoperative brain shift and should not be directly compared to patient data. In future studies, incorporating a test–retest dataset in healthy volunteers could provide additional insights into the intrinsic variability of our results, thereby strengthening confidence in the selected tractography parameters.

The next step toward achieving our goal is to test our fiber tractography approach on a larger group of pPFT patients and further investigate the morphological changes in the DRTT, before and during surgery, in order to better understand the differences in neurological outcome postoperatively. Additionally, including the premotor cortex and prefrontal cortex, along with the primary motor cortex, as inclusion regions for the thalamocortical portion of the DRTT reconstruction could be beneficial. This could provide a more comprehensive representation of the DRTT and better align with the neurological symptoms associated with disturbance of the DRTT during surgery. Taken together, our findings show that it is possible to reconstruct the DRTT in dMRI data acquired before and during surgery using this tractography protocol from most pPFT patients.

## Supplementary Information

Below is the link to the electronic supplementary material.

Supplementary file1 (DOCX 8044 KB)

Supplementary file2 (DOCX 1839 KB)

## Data Availability

The datasets generated and/or analysed during the current study are not publicly available due to patient privacy given the small number of patients but are available from the corresponding author on reasonable request.

## Abbreviations

pPFT patient: Pediatric posterior fossa tumor patient
dMRI: Diffusion magnetic resonance imaging
DRTT: Dentato-rubro-thalamic tract
ioMRI: Intraoperative magnetic resonance imaging
T1w: T1-weighted
DTI: Diffusion tensor imaging
SNR: Signal-to-noise ratio
FOD: Fiber orientation distribution
