# Supplementary material for: Evaluation of tractography parameters for dentato-rubro-thalamic tract reconstruction during pediatric posterior fossa tumor surgery
Source: MAGMA. 2025 Sep 30;39(2):275–88. doi: 10.1007/s10334-025-01297-5 (PMC13124821; doi:10.1007/s10334-025-01297-5)
Supplement: Supplementary file 1 — Supplementary file1 (DOCX 8044 KB) [file 10334_2025_1297_MOESM1_ESM.docx]

## **Appendix 1: supplementary results**

### Methods

To investigate the bimodal density distribution of the ipsilateral dentate-rubro-thalamic tract (DRTT) reconstruction based on preoperative data, we calculated the relative tumor volume. This was defined as the ratio of tumor volume to the total brain gray and white matter for each patient. A Pearson’s correlation was performed to evaluate the relation between the relative tract volume and relative tumor volume, with statistical significance set at a p-value of 0.05.

### Results

The correlation analysis shows that there is a significant correlation between the relative DRTT tract volume and the tumor volume of the patients (Fig.1, p<0.05, r = -0.61). Small tract volumes seem to be correlated with larger relative tumor volumes. **
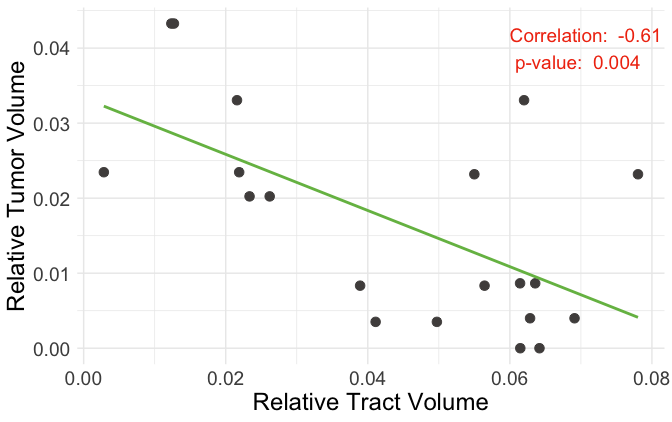
**

**Fig. 1. Correlation between relative non-decussating dentate-rubro-thalamic tract volume and tumor volume.** The tract volumes are based on preoperative patient data.

##

## **Appendix 2: supplementary figures**


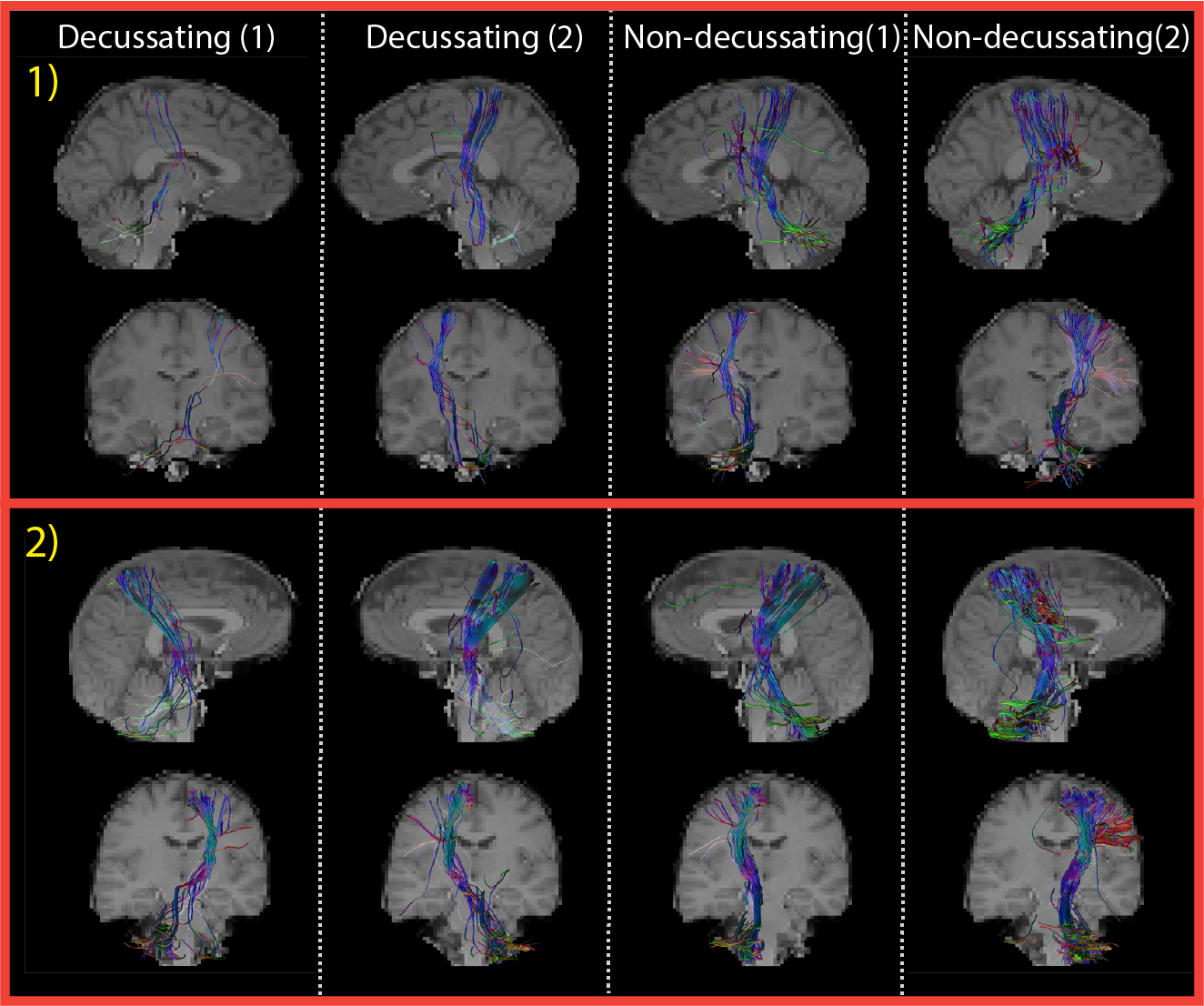


**Figure 2. Dentate-rubro thalamic tract reconstructions of healthy volunteers with optimal settings.**


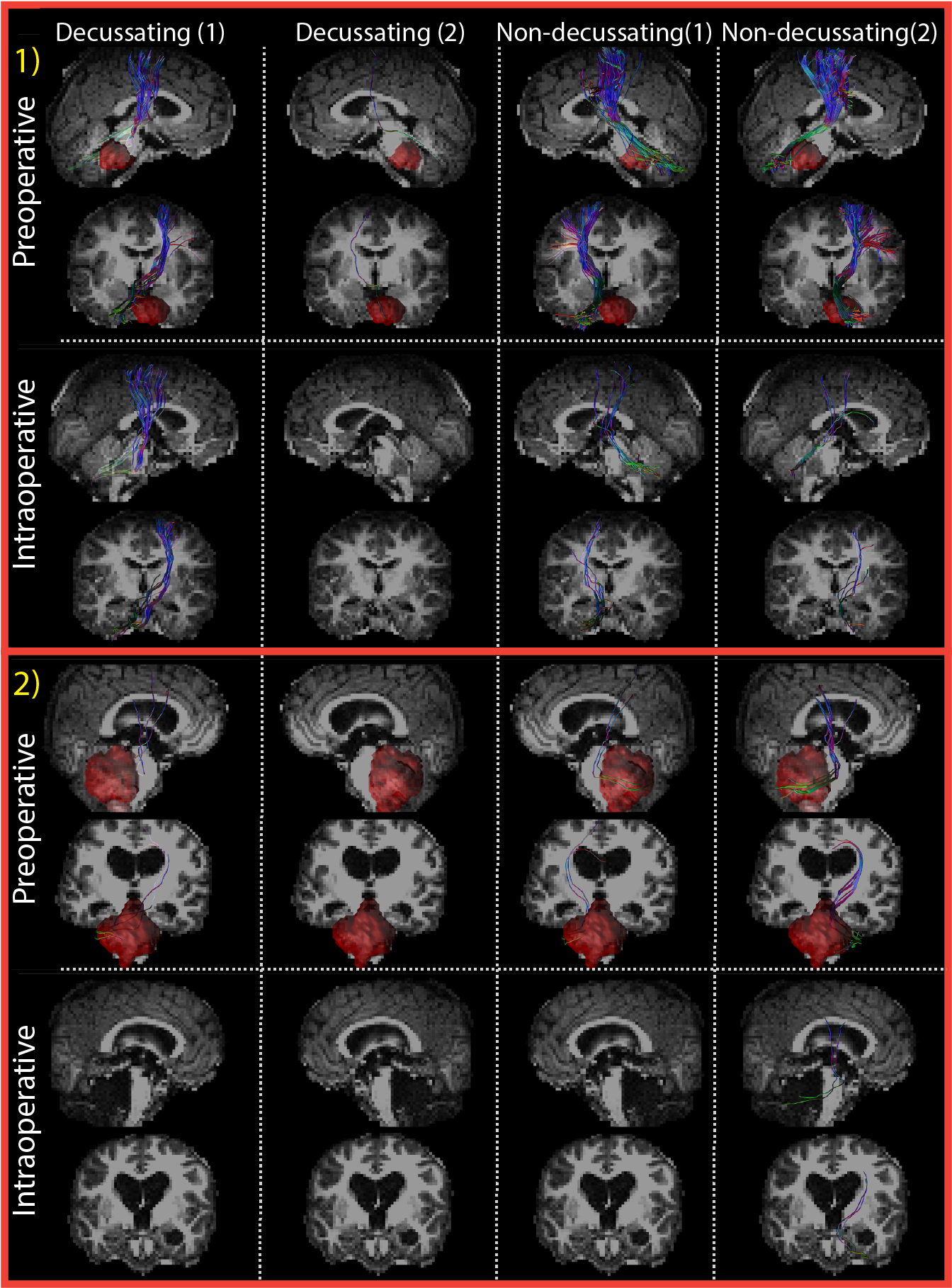


**Figure 3. Dentate-rubro thalamic tract reconstructions of patients 1 and 2, with optimal settings.**


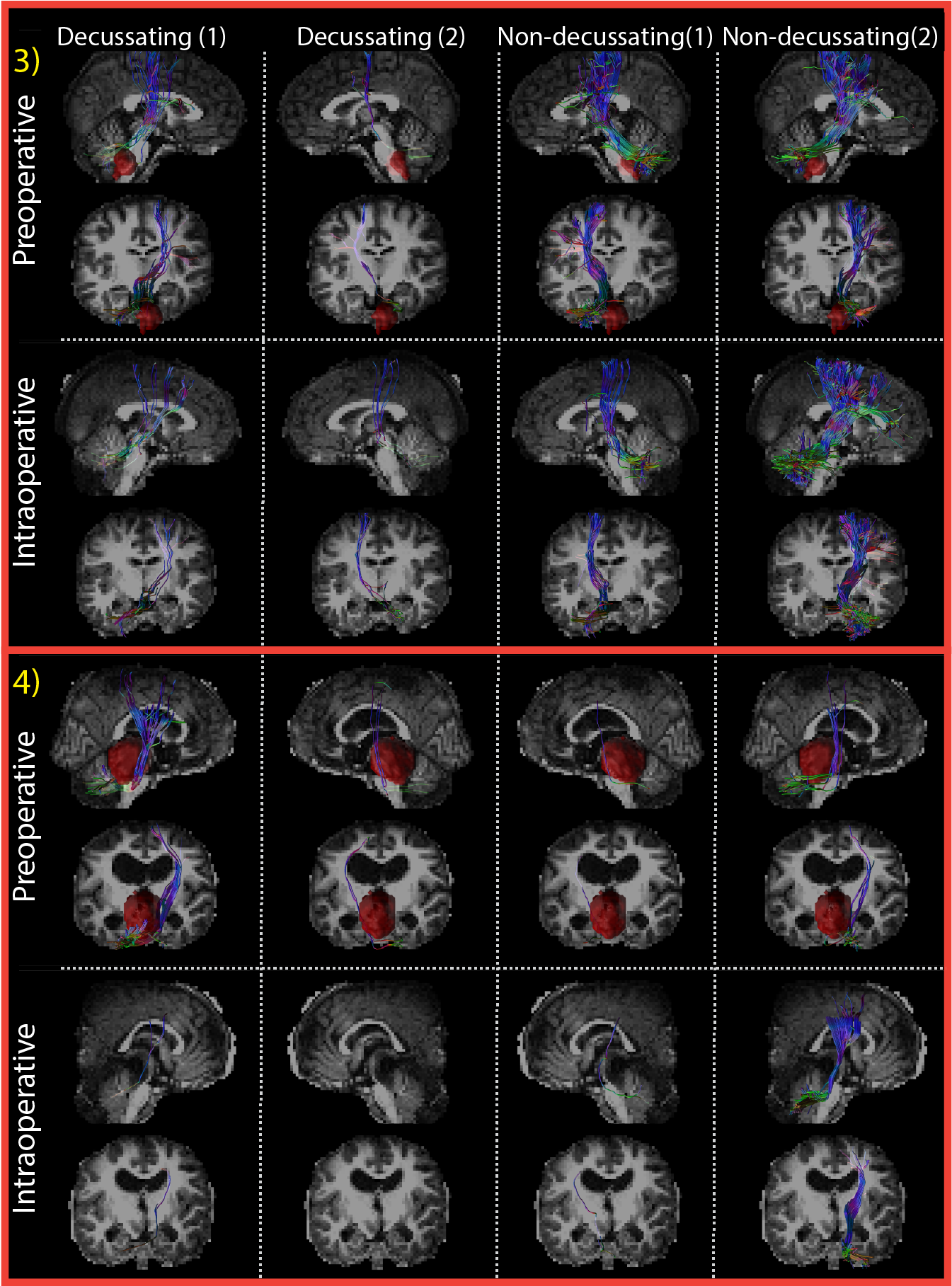


**Figure 4. Dentate-rubro thalamic tract reconstructions of patients 3 and 4, with optimal settings.**


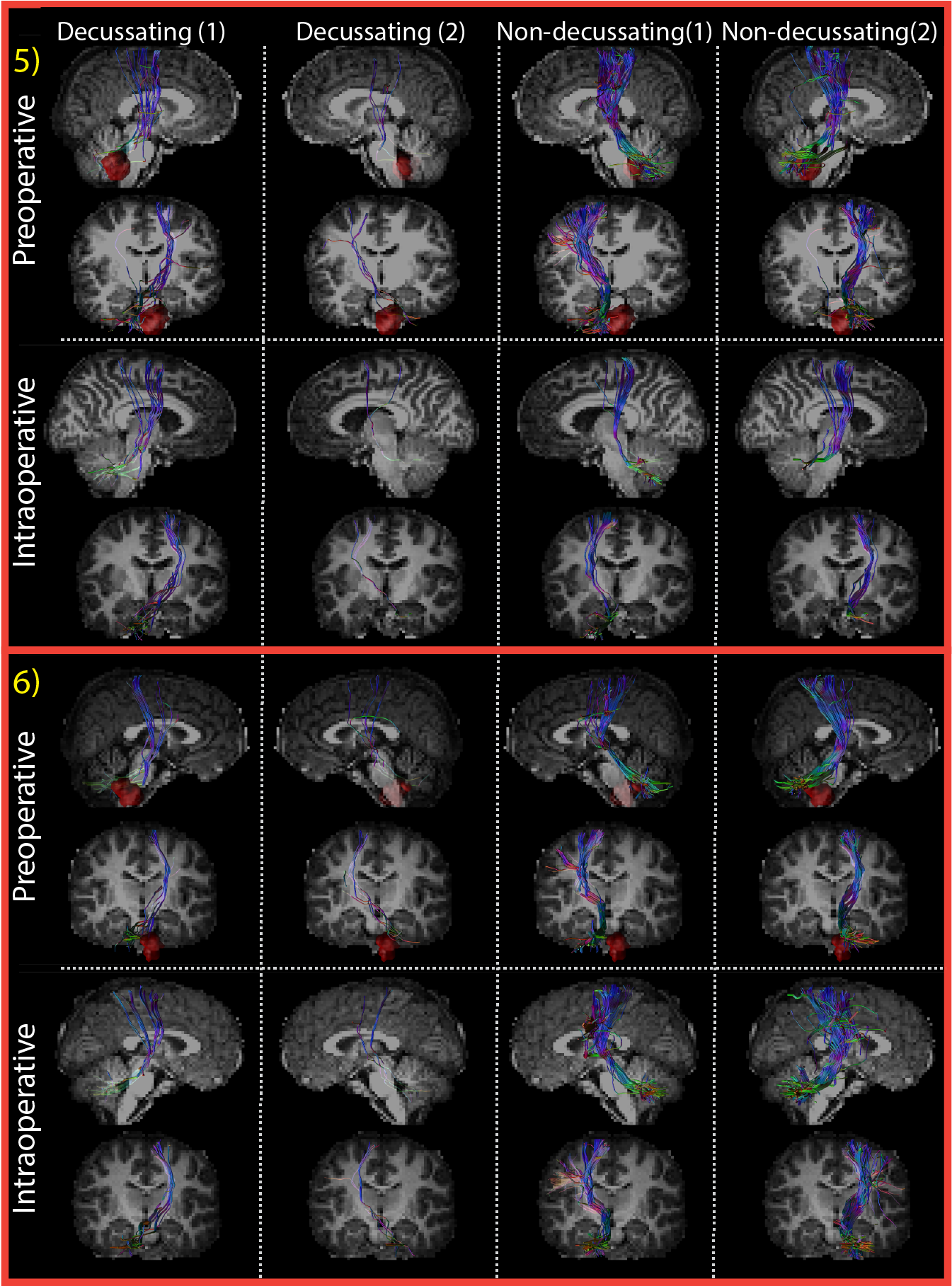


**Figure 5. Dentate-rubro thalamic tract reconstructions of patients 5 and 6, with optimal settings.**


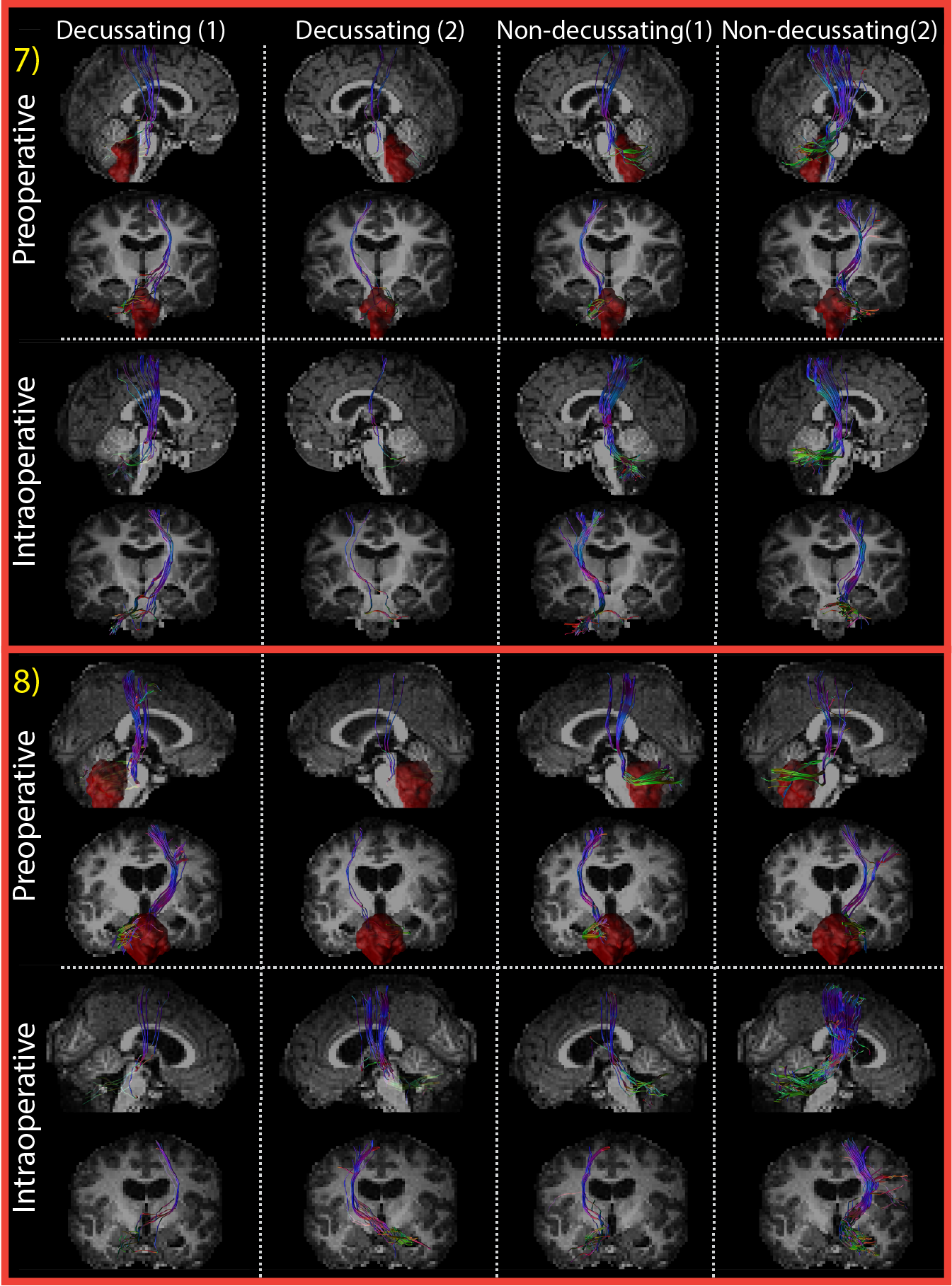


**Figure 6. Dentate-rubro thalamic tract reconstructions of patients 7 and 8, with optimal settings.**


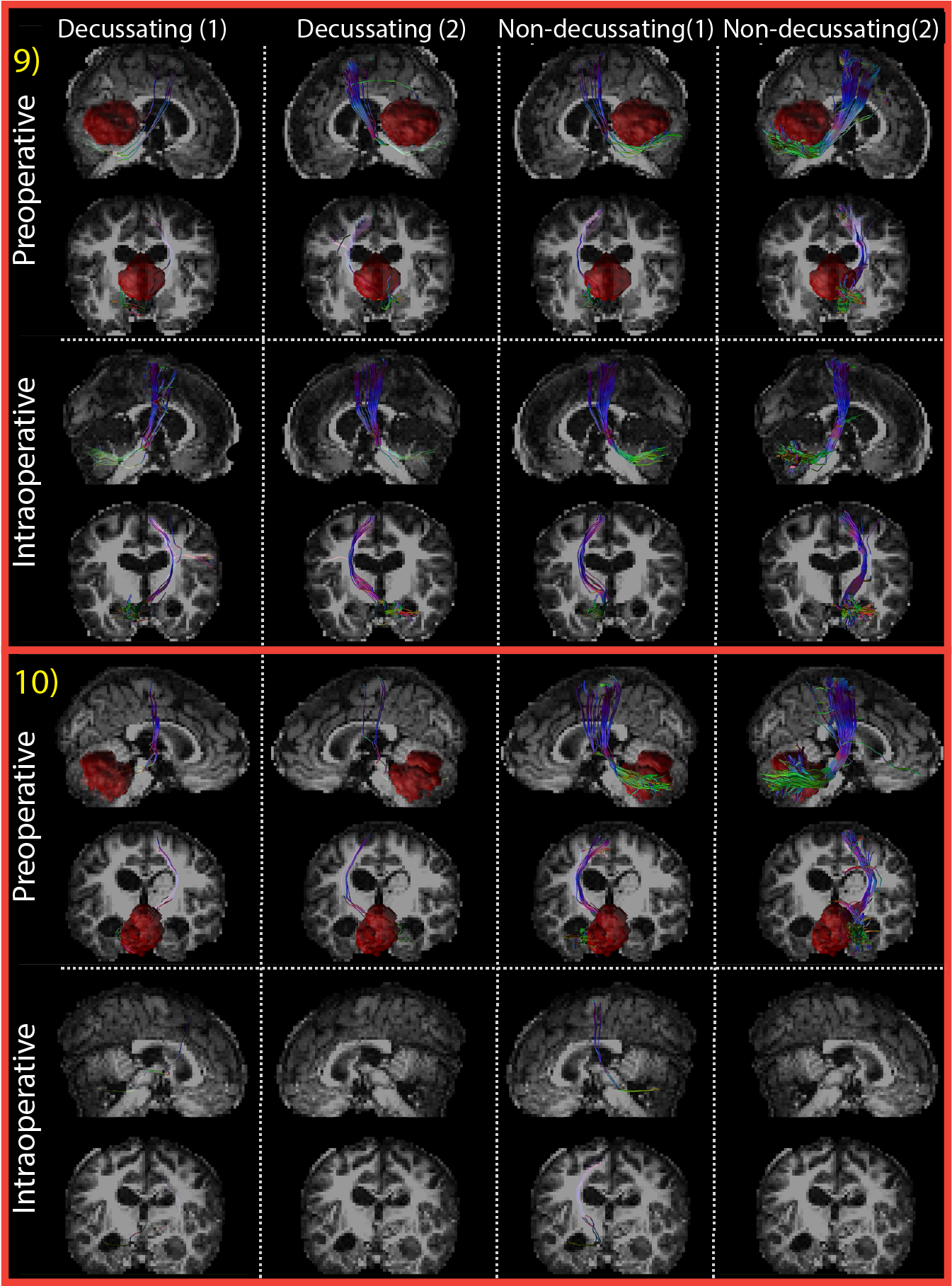


**Figure 7. Dentate-rubro thalamic tract reconstructions of patients 9 and 10, with optimal settings.**


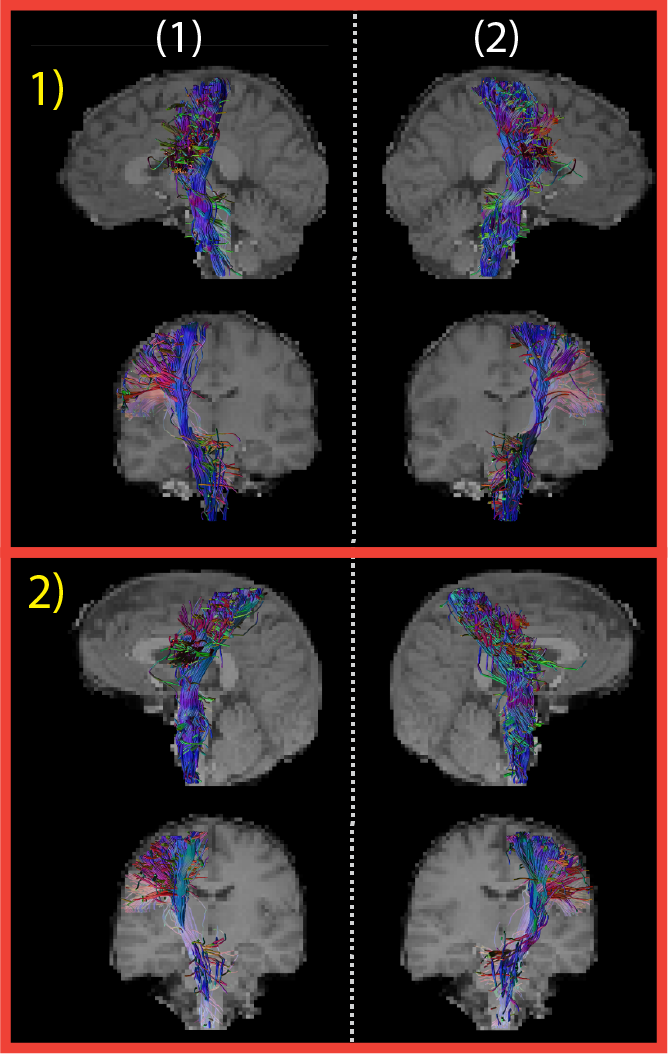


**Figure 8. Corticospinal tract reconstructions of healthy volunteers 1 and 2, with optimal settings**. The lighter-colored tracts run behind the coronal plane.


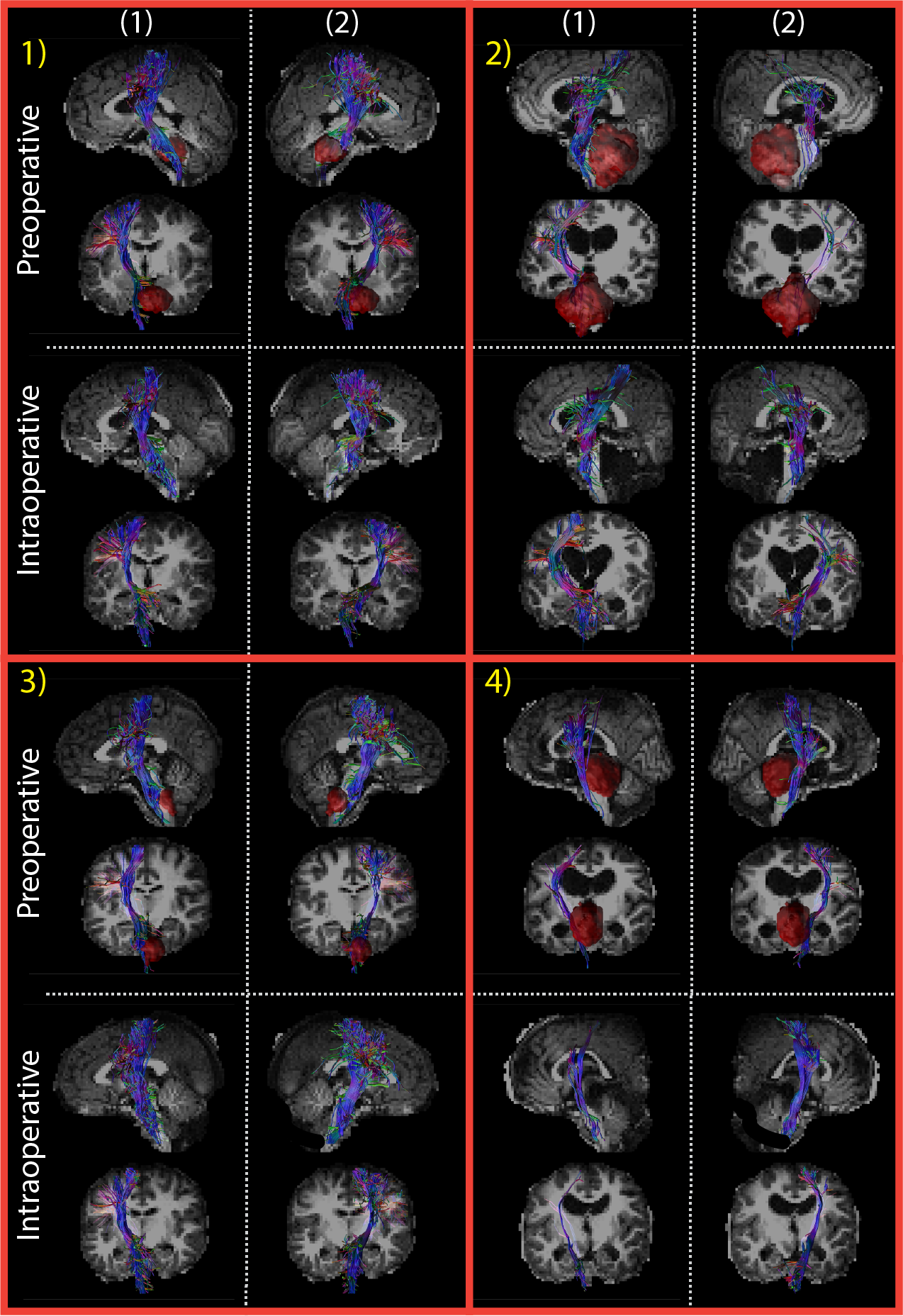


**Figure 9. Corticospinal tract reconstructions of patients 1 till 4, with optimal settings.** The lighter-colored tracts run behind the coronal plane.

**
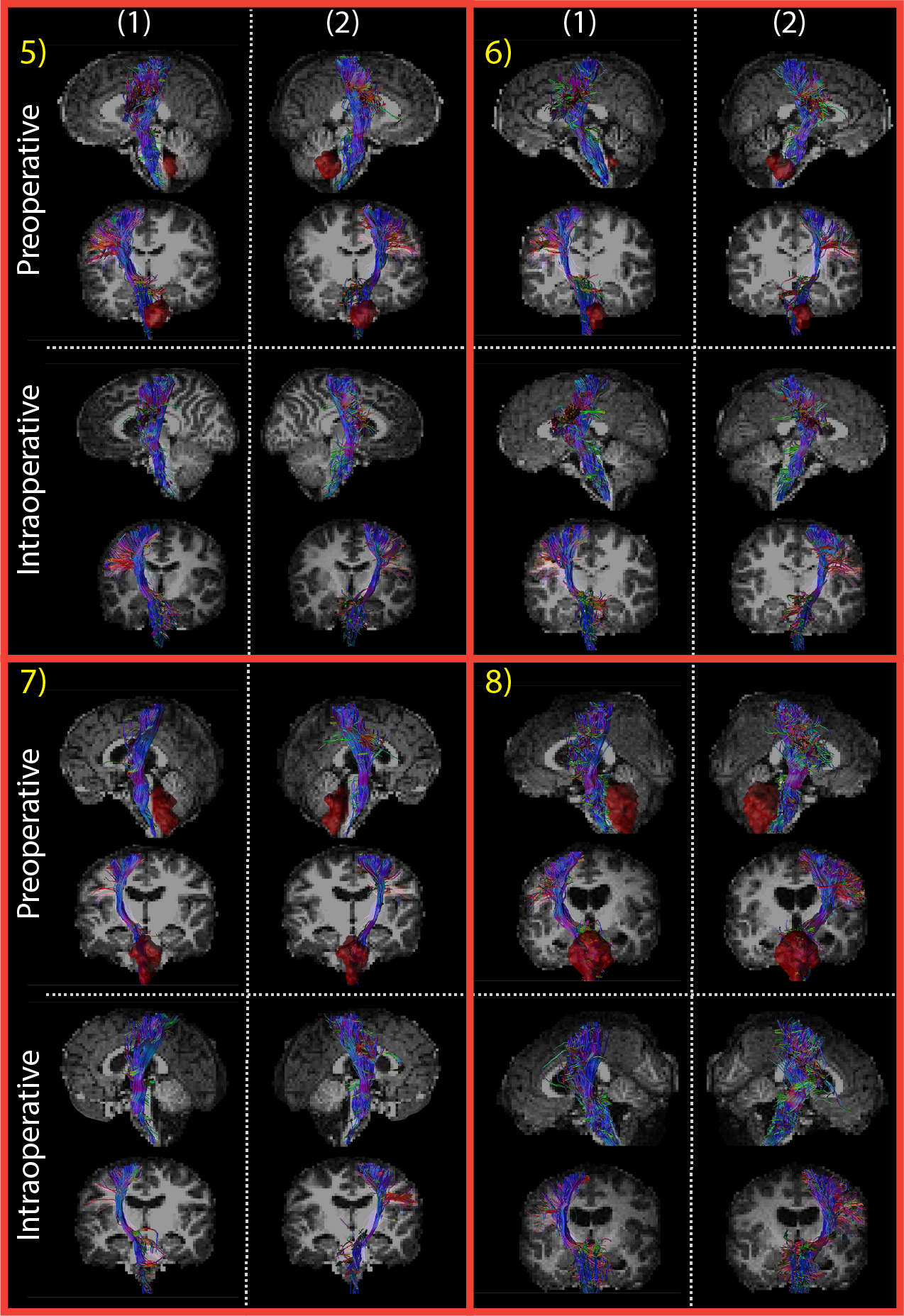
**

**Figure 10. Corticospinal tract reconstructions of patients 5 till 8, with optimal settings.** The lighter-colored tracts run behind the coronal plane.


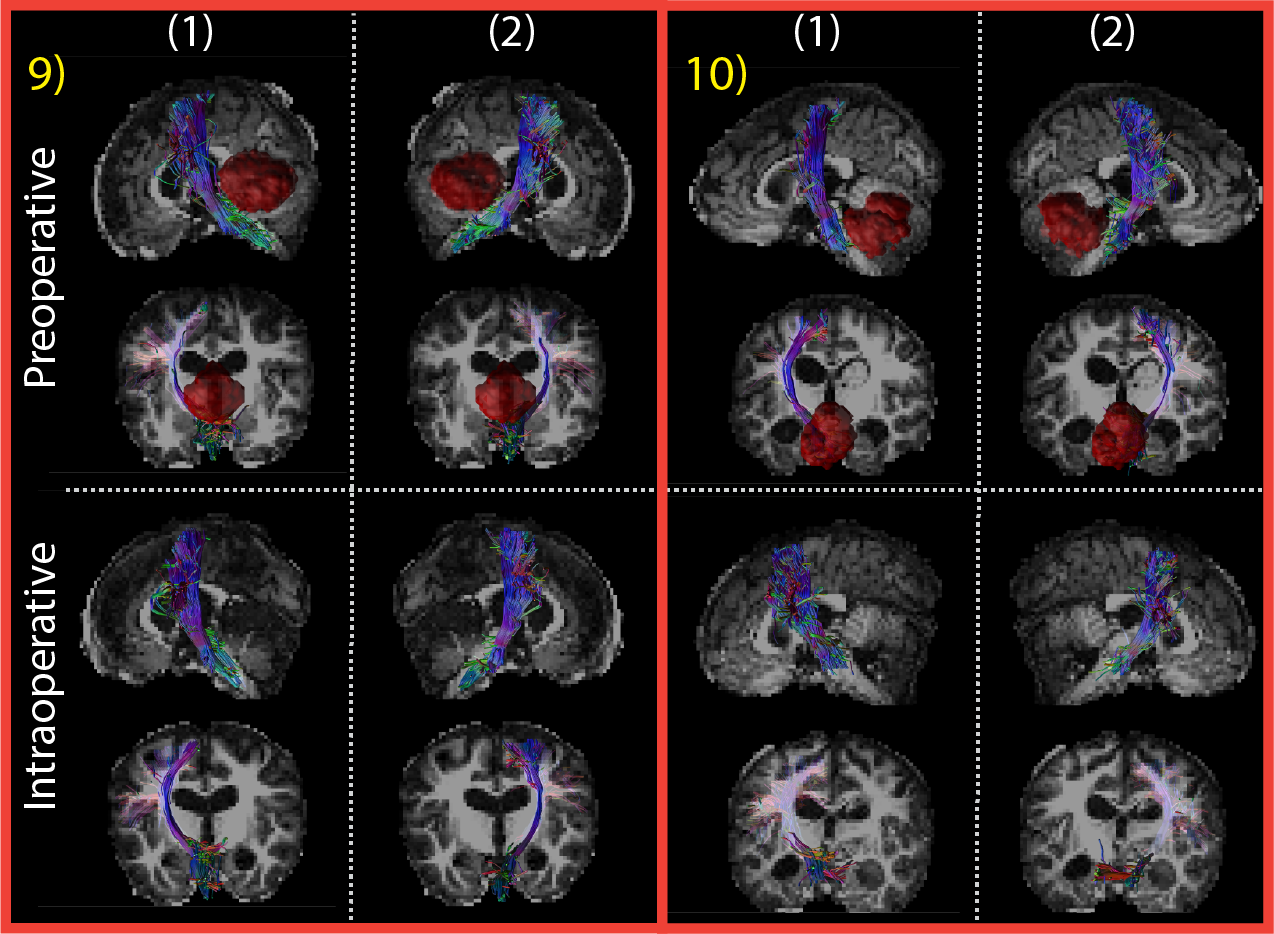


**Figure 11. Corticospinal tract reconstructions of patients 9 and 10, with optimal settings.** The lighter-colored tracts run behind the coronal plane.
