# Supplementary material for: Evaluation of tractography parameters for dentato-rubro-thalamic tract reconstruction during pediatric posterior fossa tumor surgery
Source: MAGMA. 2025 Sep 30;39(2):275–88. doi: 10.1007/s10334-025-01297-5 (PMC13124821; doi:10.1007/s10334-025-01297-5)
Supplement: Supplementary file 2 — Supplementary file2 (DOCX 1839 KB) [file 10334_2025_1297_MOESM2_ESM.docx]

**Appendix 3: Region of interest results**


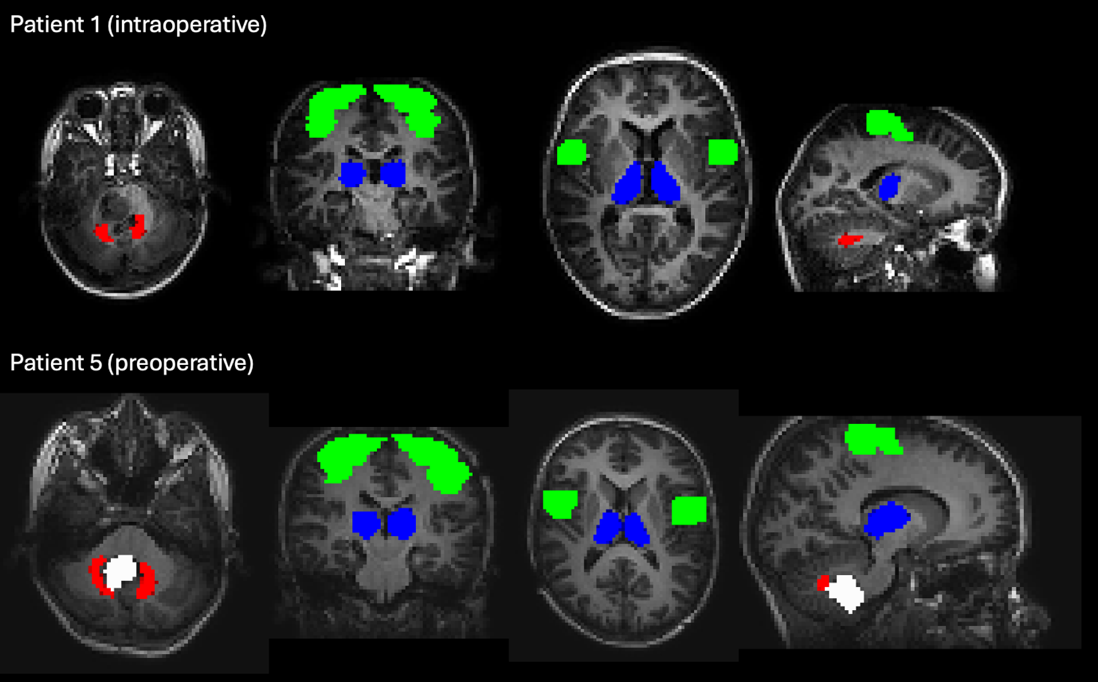

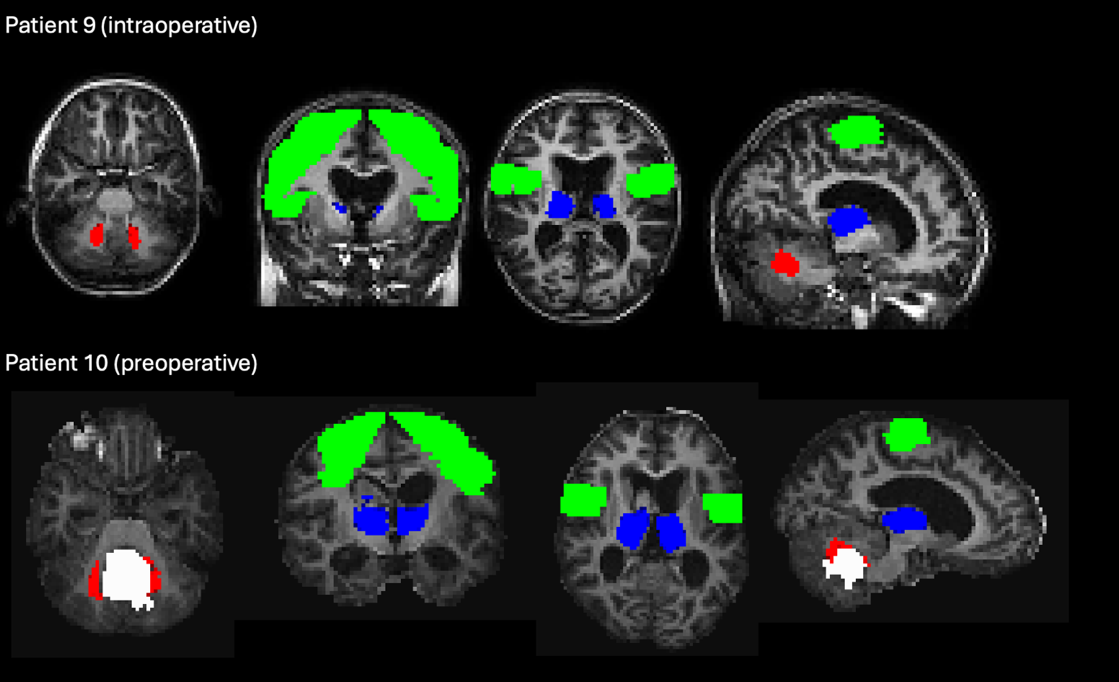


**Figure 1. Illustrations of segmented regions of interest used for reconstruction of the dentate-rubro thalamic tract**. Pre- and intraoperative examples of inclusion (AND) and exclusion regions (NOT) are shown, overlaid on a T1-weighted anatomical scan registered to diffusion space (with 2.5 mm isotropic voxels). The dentate nuclei (AND) are shown in red, primary motor cortex (AND) in green, thalamus (AND) in blue, and tumor (NOT) in white.

**Appendix 4: Scoring criteria of qualitative analysis**

Subject number:

|  | Fill out 1 option that is best suited for the question (A till H) | | | | | | | |
| --- | --- | --- | --- | --- | --- | --- | --- | --- |
| DRTT reconstruction | Ipsilateral (1)^[[1]](#footnote-1)^ | | Ipsilateral (2) | | Contralateral (1) ^2^ | | Contralateral (2) | |
| Scan session | pre | intra | pre | intra | pre | intra | pre | intra |
| Concordance with neuroanatomy | | | | | | | | |
| Which option of the reconstructed fiber tracts corresponds best to known anatomical landmarks or structures? | … |  |  |  |  |  |  |  |
| Trustworthiness  Which option …. (here you can fill out multiple suitable options per question) | | | | | | | | |
| … has a disturbing amount of false positive streamlines? Unclear what pathway can be seen? | … |  |  |  |  |  |  |  |
| …has some false positive outlier streamlines, but the majority is in agreement with the expected trajectory? | … |  |  |  |  |  |  |  |
| … seems to mainly follow the expected trajectory, but some streamlines could be missing (slight underrepresentation of the tract)? | … |  |  |  |  |  |  |  |
| … is missing most streamlines of the tract? | … |  |  |  |  |  |  |  |
| … is missing all streamlines? | … |  |  |  |  |  |  |  |
| Overall | | | | | | | | |
| Based on all questions above, which option would you rate as the most trustworthy reconstruction of the DRTT? | … |  |  |  |  |  |  |  |
| How would you rate the overall quality^3^ between 1 and 10 of the reconstruction, as chosen above, for clinical practice? ^4^ | … |  |  |  |  |  |  |  |

**Table 1. Scoring criteria form used for qualitative analysis.** This form was filled out for each subject by both members of the anatomical expert panel who were blinded for the parameter combinations (A till H) that were shown in a randomized order. The final answer on the overall most trustworthy reconstruction was used for further analysis. Both sides of the ipsi- and contralateral DRTT reconstructions were shown for each subject of their pre- and intraoperative datasets.

**Appendix 5: Fiber tractography adaptation to anatomical displacement**


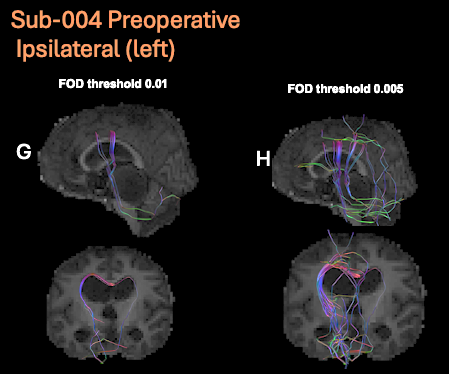


**Figure 2. Individual adaptation of fiber tractography parameters to anatomical displacement**. Panels G and H illustrate variations in streamline reconstruction of the left non-decussating DRTT of patient 4, who presented with hydrocephalus at the preoperative timepoint. The anatomical displacement caused by the hydrocephalus results in a limited number of reconstructed streamlines when applying the optimized settings for the study population (G: angle threshold 60°, FOD threshold 0.01). Relaxing the FOD threshold to 0.005 (H) increases spatial coverage, but at the prize of many false positives streamlines which require further removal.

**Appendix 6: Similar reconstructions with differing threshold settings**
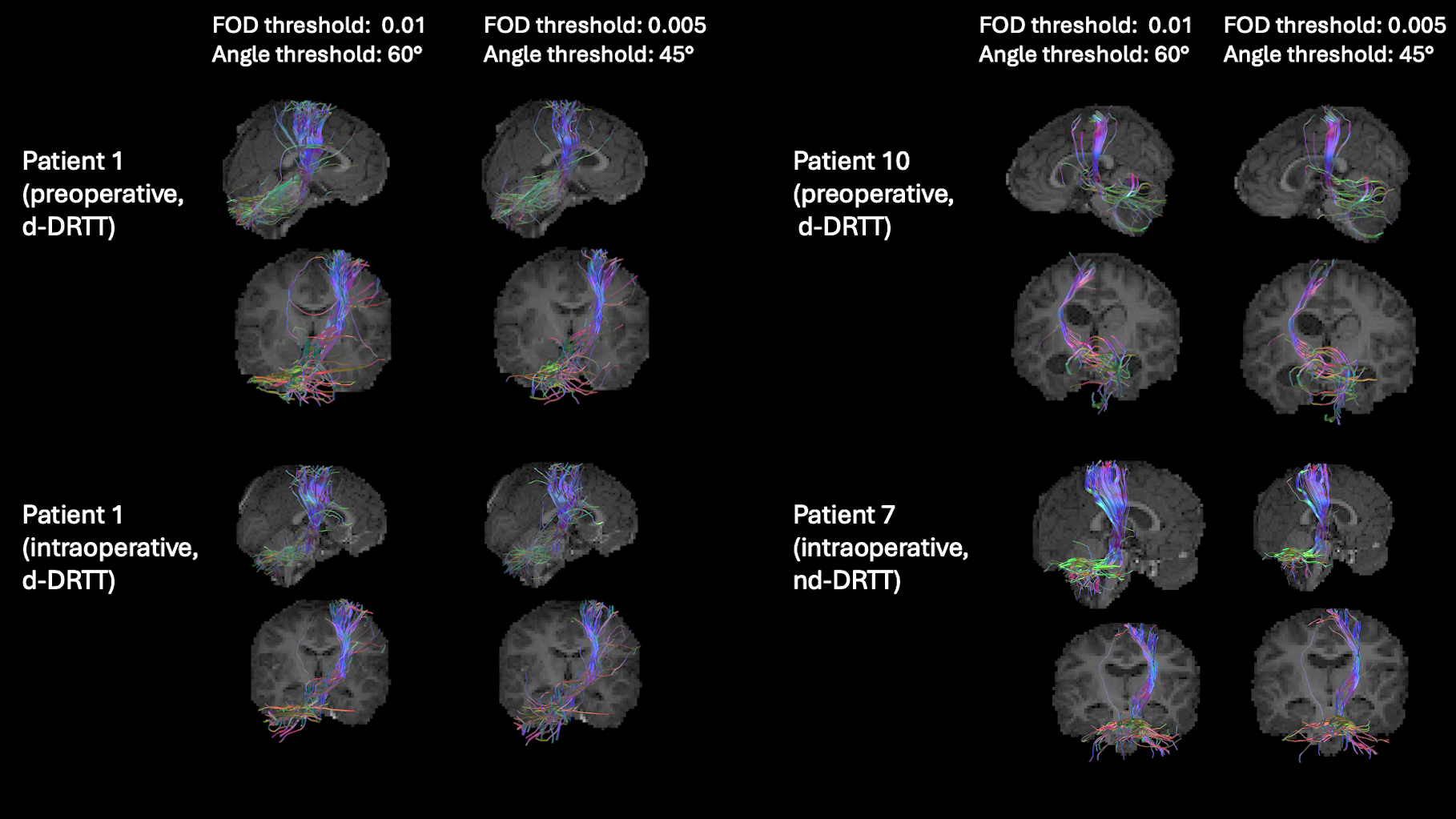


**Figure 3. Examples of similar reconstructions with differing threshold settings.** Pre- and intraoperative examples of patients are shown to illustrate dentato-rubro-thalamic tract (DRTT) reconstructions of comparable quality, yielded with differing parameter combinations. FOD = fiber orientation distribution, d-DRTT = decussating DRTT, nd-DRTT = non-decussating DRTT.

**Appendix 7: Repetition time values of all patients and measurements**

|  | Preoperative TR (ms) | | | Intraoperative TR (ms) | | |
| --- | --- | --- | --- | --- | --- | --- |
| Patient | 1) B1000 | 2) B2000 | 3) Rev. enc | 1) B1000 | 2) B2000 | 3) Rev. enc |
| 1 | 7724 | 7724 | 7724 | 7724 | 7724 | 7724 |
| 2 | 7724 | 7724 | 7724 | 7734 | 7734 | 7734 |
| 3 | 7733 | 7733 | 7733 | 7731 | 7731 | 7731 |
| 4 | 7724 | 7723 | 7724 | 7724 | 7723 | 7724 |
| 5 | 7723 | 7723 | 7723 | 7721 | 7721 | 7721 |
| 6 | 7723 | 7723 | 7723 | 7723 | 7723 | 7723 |
| 7 | 7720 | 7720 | 7720 | 7720 | 7720 | 7720 |
| 8 | 7723 | 7723 | 7723 | 7723 | 7723 | 7723 |
| 9 | 7723 | 7723 | 7723 | 7723 | 7723 | 7723 |
| 10 | 7723 | 7723 | 7723 | 7723 | 7723 | 7723 |

**Table 2. Repetition time values of all patients and measurements**. The repetition time (TR) was set on a range from 7000 to 8200 ms for all diffusion MRI acquisitions to allow for variability in head orientation and to ensure thermal safety. Acquisitions numbers one till three correspons to the separate diffusion MRI acquisitions described in table 2 of the manuscript with the respective imaging parameters.

1. Non-decussating DRTT

   ^2^ Decussating DRTT

   ^3^ Overall morphological quality in the context of known anatomy

   ^4^ From the perspective of neurosurgical or neuro(-onco)logical practice [↑](#footnote-ref-1)
